# Both the scion and rootstock of grafted grapevines influence the rhizosphere and root endophyte microbiomes, but rootstocks have a greater impact

**DOI:** 10.1186/s40793-024-00566-5

**Published:** 2024-04-23

**Authors:** Vincent Lailheugue, Romain Darriaut, Joseph Tran, Marine Morel, Elisa Marguerit, Virginie Lauvergeat

**Affiliations:** 1 EGFV, Univ. Bordeaux, Bordeaux Sciences Agro, INRAE, ISVV, Villenave d’Ornon, F-33882 France; 2 Present Address: Univ Rennes, CNRS, ECOBIO (Ecosystèmes, biodiversité, évolution) - UMR 6553, Rennes, F-35000 France

**Keywords:** Bacteria, Fungi, Arbuscular mycorrhizal fungi, Root system, Metabarcoding, PICRUSt2, FUNGuild

## Abstract

**Background:**

Soil microorganisms play an extensive role in the biogeochemical cycles providing the nutrients necessary for plant growth. Root-associated bacteria and fungi, originated from soil, are also known to influence host health. In response to environmental stresses, the plant roots exude specific molecules influencing the composition and functioning of the rhizospheric and root microbiomes. This response is host genotype-dependent and is affected by the soil microbiological and chemical properties. It is essential to unravel the influence of grapevine rootstock and scion genotypes on the composition of this microbiome, and to investigate this relationship with plant growth and adaptation to its environment. Here, the composition and the predicted functions of the microbiome of the root system were studied using metabarcoding on ten grapevine scion-rootstock combinations, in addition to plant growth and nutrition measurements.

**Results:**

The rootstock genotype significantly influenced the diversity and the structure of the bacterial and fungal microbiome, as well as its predicted functioning in rhizosphere and root compartments when grafted with the same scion cultivar. Based on β-diversity analyses, 1103P rootstock showed distinct bacterial and fungal communities compared to the five others (RGM, SO4, 41B, 3309 C and Nemadex). The influence of the scion genotype was more variable depending on the community and the investigated compartment. Its contribution was primarily observed on the β-diversity measured for bacteria and fungi in both root system compartments, as well as for the arbuscular mycorrhizal fungi (AMF) in the rhizosphere. Significant correlations were established between microbial variables and the plant phenotype, as well as with the plant mineral status measured in the petioles and the roots.

**Conclusion:**

These results shed light on the capacity of grapevine rootstock and scion genotypes to recruit different functional communities of microorganisms, which affect host growth and adaptation to the environment. Selecting rootstocks capable of associating with positive symbiotic microorganisms is an adaptation tool that can facilitate the move towards sustainable viticulture and help cope with environmental constraints.

**Supplementary Information:**

The online version contains supplementary material available at 10.1186/s40793-024-00566-5.

## Background

Grapevine is a cultivated perennial plant of major economic interest. In 2022, the International Organization of Vine and Wine estimated the world vineyard surface area to be 7.3 millions of hectares, producing 258 millions of hectolitres of wine with a world trade volume of 107 mhl, representing €37.6 bn. Since the phylloxera crisis at the end of the 19th century, *Vitis vinifera* has mostly been cultivated by grafting onto species or hybrids of American *Vitis* rootstock [1]. Market globalization and the rapid industrialization of agricultural sectors have introduced new requirements for winegrowers. Sustainable agriculture means limiting the use of chemical fertilizers and pesticides, while maintaining yields and berry quality. In addition, global warming is causing higher temperatures and radiation, as well as changes in precipitation patterns, resulting in severe drought periods that have negative impacts on vine development [2]. Viticulture adaptation is currently based on the modification of cultivation practices and/or the plant material such as the rootstock or scion genotypes [3, 4].

Plants closely interact with various microorganisms such as bacteria, fungi, archaea, protists, or viruses, known as plant microbiota. It is now well established that microbial communities play indisputable roles in supporting grapevine health and adaptation to environmental conditions [5, 6]. This awareness led to the emergence of the “holobiont” concept, which considers the multicellular host and its associated microbiota as a functional entity [7]. In the rhizosphere, defined as the area of soil immediately surrounding the roots, several microorganisms interact, directly or not, with the root system. Some of them can reach the root endosphere through intercellular junctions in the epidermis or wounds on the rhizoplane [8]. On one hand, soil microorganisms can be detrimental for grapevine longevity and productivity. For example, pathogenic fungi cause trunk diseases such as Esca complex, Phomopsis, Eutypa or Botryosphaeriae dieback in established vineyards, as well as Petri disease or Black foot in young vineyards [9]. On the other hand, several microorganisms provide benefits to the plant in terms of biofertilization, biostimulation and biocontrol properties. For instance, plant growth promoting rhizobacteria (PGPR) can increase plant nutrient uptake by solubilizing non-assimilable forms of phosphorus into plant-assimilable forms or by fixing atmospheric nitrogen [10]. Other PGPR can interfere with phytohormone homeostasis or synthetize pseudo-phytohormones, promoting plant growth and adaptation to environmental stresses [11]. Mechanisms used by bacteria to counter pathogens are antibiosis, suppression of virulence factors, niche competition and activation of the plant-induced systemic resistance [12]. As reviewed by Trouvelot et al. (2015), viticulture adaptation can benefit from arbuscular mycorrhizal fungi (AMF). They improve grapevine development by increasing access to soil nutrients and regulation of plant nutrient transporters, primarily for phosphorus and nitrogen. These root symbionts can increase grapevine tolerance to drought, iron deficiency in calcareous soil, soil salinity or heavy metals. Besides abiotic stresses, AMF also confer tolerance to biotic stressors thanks to the mycorrhiza-induced resistance (MIR) and/or the systemic acquired resistance (SAR) [13].

In response to the environment, plant roots exude a wide range of chemical compounds into the rhizosphere, including small molecules (e.g., organic acids, sugar, aliphatic acids, fatty acids, amino acids, flavonoids, and secondary metabolites) and complex molecules (e.g., proteins and mucilage) [14]. Rhizodeposits influence the biology of microorganisms in the rhizosphere as a source of carbon, antimicrobial or chemoattractant compounds [15]. Plant hormones are also involved in plant-microorganism interactions, such as strigolactones, which have been reported to stimulate hyphal branching in AMF [16]. The composition of root exudate is mainly influenced by edaphic (properties, nutrients, indigenous microorganisms) and plant factors (species, phenophase, root morphology), including host genotype [17]. Selecting genotypes with a low capacity for association with pathogenic organisms, or conversely, with high capacity for association with symbiotic organisms, could help overcome some of the challenges faced in viticulture.

Few studies have been published in recent years on the effects of grapevine rootstock genotype on root and/or rhizosphere microbiota composition. Analyses of metabarcoding data targeting bacterial communities only [18–21] or bacterial and fungal communities [22–25] show that rootstock genotype influences the rhizosphere microbiota diversity and community structure. Marasco et al. (2022) further displayed that this genotype-dependent effect was also present for the bacterial and fungal communities of the root endosphere [24]. In their study, seven graft/stock combinations from ten vineyards in two geographical areas of Italy were analyzed. The results show that while the major factors driving microbial community structure are soil type and cultivation practices, the interaction of the two genotypes is the second driver, even more than each genotype separately. These results confirm those obtained by Vink et al. (2021), who studied bacterial communities in the rhizosphere of four cultivars combined with four rootstocks in the same vineyard [26]. A recent study showed that root bacterial communities are influenced by rootstock genotypes in a site-specific manner [27]. Given that soil quality is the most significant factor influencing the microbiota of viticultural soils [27, 28] and therefore that of the root system, it is very difficult to compare the structure and function of the microbiota of different genotypes between all these studies. Finally, only one study investigated the AMF communities of grapevine roots from three vineyards and nine rootstocks by denaturing gradient gel electrophoresis (DGGE) and trap cultures [29]. The authors demonstrated that the rootstock genotype significantly influenced the AMF community colonizing the roots.

The current aim is to explore the greatest possible genetic variability of grapevine rootstock or scion genotypes, grown on the same plot, to identify those capable of recruiting the most effective functional microbiota and to test the influence of scion cultivars on these responses. This also requires further knowledge of the links between community structure and biological function, and the potential impact on vine growth and resistance. First, we quantified the level of rhizospheric bacteria and fungi using qPCR and cultivable approaches. Secondly, the communities of bacteria, fungi, and AMF in the rhizosphere and the root endosphere were explored using Illumina sequencing of the 16S rRNA gene, ITS and 28S rRNA gene, respectively. Finally, the functions of bacterial and fungal microbiome were predicted using PICRUSt2 and FUNGuild respectively, and microbial variables measured in both microbiomes were correlated to plant phenotypic traits and mineral nutrition.

## Materials and methods

### Plot, plant material and phenotyping

The experiment was set up in Bordeaux, France (44°47’26.6"N 0°34’26.7"W), in GreffAdapt plot, a large-scale experimental plot designed with 55 rootstocks grafted with five scions, divided into three blocks of five vines per combination, i.e., 15 vines per combination, distributed randomly within each block [30]. The climate is oceanic, with mild winters and high rainfall. The study was carried out in May 2021. The plot was planted with a density of 6,666 vines/ha and is pruned using the simple Guyot method and trained with a vertical trellis system. A natural cover crop is planted on each row and is mowed according to grass growth. The inter-rows are not tilled, but the area under the vines is tilled mechanically. Fungicide treatments are applied regularly to limit the development of downy and powdery mildew during the vegetative period. The plot is not irrigated. Phenotypic monitoring was carried out annually on the vines. This included the number of shoots, fertility (number of bunches per shoot), winter pruning weight and the vigor conferred (pruning weight / number of shoots). The number of bunches, berry yield and δ^13^C of berry juice were assessed at the harvest, when the berries from the different scions were at maturity (between August and October). Mineral content in the petioles (collected between the fourth and sixth leaf of the shoot on the cane side) and the roots were assessed by Waypoint Analytical (Virginia, USA). We selected six rootstocks’ genotypes, presenting contrasting drought tolerance and vegetative vigor conferred to the scion (Additional file 2: Table S1), grafted with Cabernet-Sauvignon (CS) clone 169, to study the impact of the rootstock genotype on the rhizosphere and root endophyte microbiomes: 1103 Paulsen (1103P), 3309 Couderc (3309 C), 41 B Millardet et de Grasset (41 B), Nemadex Alain Bouquet (Nem), Riparia Gloire de Montpellier (RGM) and Selection Oppenheim 4 (SO4). To evaluate the effect of the scion, we focused on one of these rootstocks (RGM) grafted with the five grape varieties: CS, Grenache (Gre), Pinot noir (PN), Syrah (Syr) and Ugni blanc (UB) (Additional file 2: Table S1). Six individuals were sampled for each scion-rootstock combination, three on block 2 and three on the block 3. Blocks 2 and 3 correspond to different areas on the plot designed according to the soil resistivity. Aurea Agrosciences (Orléans, France) assessed the soil properties and defined them as sandy gravelly soil in both blocks (Additional file 2: Table S2).

### Sample collection and processing

Roots from different parts of the root system with attached soil particles were collected at a depth of 20–30 cm in sterile tubes containing 40 ml of 0.85% NaCl, placed in a cooler before being processed in the laboratory. To separate the rhizosphere from the roots, the samples were vortexed for 5 min and then centrifuged at 4000 g for 5 min. The roots were transferred into a new tube before discarding the supernatant to keep only the rhizosphere. One gram of rhizosphere was diluted into 9 ml of physiological solution for the plating approach and the rest was lyophilized and stored at -80 °C before DNA extraction. To investigate endophyte microorganisms, root tissues were surface sterilized by soaking in 3% sodium hypochlorite (NaClO) for 1 min, followed by 3% H2O2 for 1 min and finally by washing with sterile distilled water five times. Sterilized roots were ground in 35-ml stainless-steel grinding jars with 20-mm stainless steel balls at 30 oscillations per second for 30 s with the mixer mill MM400 (Retsch, Haan, Germany), using liquid nitrogen and then stored at -80 °C until DNA extraction.

### Quantification of cultivable bacterial and fungal colonies

The rhizosphere was vortexed, and the supernatant was serially diluted in 0.85% NaCl to 1/100 for the fungi and to 1/10 000 for the bacteria before plating 100 µl of each dilution. Cultivable bacteria were quantified on R2A medium (0.5% yeast extract, 0.5% proteose peptone, 0.5% casamino acids, 0.5% glucose, 0.5% soluble starch, 0.3% sodium pyruvate, 0.3% H_2_KO_4_P, 0.05% MgCl_2_, pH 7) supplemented with nystatin (25 mg. l^− 1^). Cultivable fungi were quantified on Potato Dextrose Agar medium (BioKar) supplemented with 500 mg l^− 1^ of gentamicin and 50 mg l^− 1^ of chloramphenicol. Incubation was done at ambient temperature in the dark and the colony-forming units (CFUs) were counted five and seven days after fungal and bacterial plating, respectively.

### DNA extraction from roots and soil samples

DNA of root tissues was extracted from 150 mg of frozen powder using the DNeasy® Plant Mini kit (Qiagen) with a modified protocol adapted from Pouzoulet et al. (2013) [31]. At step two, the root powder was incubated for 30 min in 1 ml of CTAB buffer (2% CTAB, 2% PVPP, 1.4 M NaCl, 100 mM Tris, 20 mM EDTA) supplemented with 0.2% β-mercaptoethanol and 0.16% RNase A (100 mg. ml^− 1^). At step three, 325 µl of buffer P3 was used and the incubation step on ice was increased to 10 min. At step four, the lysate was centrifugated at 6500 g for 10 min. At step six, the AW1 buffer was replaced by the AW2 buffer. At step eleven, DNA was eluted in 50 µl of buffer AE and step twelve was deleted. DNA from the rhizosphere was extracted from 250 mg of lyophilized soil using DNeasy® PowerSoil® Pro kit (Qiagen) according to the manufacturer’s instructions with some adaptations. The vortex step was replaced by three 30-second runs, each on power 4 m. s^− 1^ with the homogenizer FastPrep®-24. At step thirteen, an extra wash was performed using 350 µL of C5 solution. At step sixteen, DNA was eluted twice into a final volume of 80 µL of C6 solution. Extracted DNA was quantified on a Qubit® 3.0 fluorometer (Thermo Fisher Scientific) using the Qubit™ dsDNA HS Assay Kit, and its quality was checked using a NanoDrop™ 2000/2000c spectrophotometer (Thermo Fisher Scientific). DNA was then stored at -20 °C until further use.

### Quantitative PCR (qPCR) amplification of bacterial and archaeal 16S and fungal 18S rRNA genes

Analyses of qPCR were performed on the DNA extracted from the rhizosphere using primers and cycling conditions described in Darriaut et al. (2021) [32]. The primer pairs 515R/341F, FR1/FF390 and Arch967F/Arch1060R were used to amplify bacterial 16S rRNA, fungal 18S rRNA and archaeal 16S rRNA genes, respectively. Reactions were carried out in a final volume of 20 µl composed of 10 µl of GoTaq® qPCR Master Mix (Promega), 1 µl of each primer (10 µM), 7 µl of nuclease-free water and 1 µl of extracted DNA (1 ng. µl^− 1^). Standards used for absolute quantification were obtained from amplicons previously subcloned into pGEM®-T easy vector system (Promega) by Darriaut et al. [32]. Each sample was quantified in triplicates using the CFX96 Touch Real-Time PCR Detection System (Bio-Rad Laboratories, France). The efficiencies of the qPCR were between 80% and 99% (R² > 0.99). All analyses included PCR negative and positive controls.

### Target metabarcoding on bacterial 16S rRNA gene, fungal ITS region and Glomeromycete 28S rRNA gene

The bacterial 16S rRNA gene was amplified using the primer pair 341 F/785R, while the fungal ITS1 region was amplified using the ITS1F/ITS2 primer pair. PCR reactions were monitored in a final volume of 25 µl composed of 5 µl of 5X GoTaq® colorless reaction buffer (Promega, France), 0.5 µl of each primer (10 µM), 0.5 µl of dNTPs (10 mM), 0.125 µl of GoTaq® G2 DNA Polymerase (Promega, France), 1 µl (at 1 ng.µl^− 1^) or 2.5 µl (at 5 ng.µl^− 1^) of DNA extracted from roots or soil respectively, and nuclease-free water (q.s.p 25 µl). The fungal 28S rRNA gene was amplified using a nested PCR approach to target the *Glomeromycota* division. A first PCR was performed using the primer pair LR1/NDL22 specific to the eukaryote 28S rRNA gene. The obtained product was diluted to 1/100th and 5 µl were used as template for the second PCR using the primer pair FLR3/FLR4 specific to *Glomeromycota*. All analyses included PCR negative and positive controls. The sequences of the Illumina adapters and the primers, as well as the cycling conditions are listed in Additional file 2: Table S3. Further steps were carried out at the Plateforme Génome Transcriptome de Bordeaux (Cestas, France). The PCR products were purified with the platform-specific SPRI magnetic beads (1X ratio) and quantified using Quant-iT™ dsDNA assay kit (ThermoFisher, France). MID and Illumina sequencing adapters were added. Libraries were pooled in equimolar amounts using a Hamilton Microlab STAR robot and sequenced on an Illumina MiSeq platform using the MiSeq Reagent Kit v2 (2 × 250 bp). Obtained sequences were demultiplexed with index search at the PGTB facility. The quality of the obtained sequences were first checked with FastQC v.0.11.8 [33]. Sequences were quality filtered, trimmed, denoised, and clustered into Operational Taxonomy Units (OTUs) using FROGS pipeline from Galaxy instance [34, 35]. This involved assembling raw forward and reverse reads for each sample into paired-ended reads with a minimum overlapping of 50 nucleotides and 0.1 mismatch using the VSEARCH tool [36]. Primers were removed using Cutadapt [37], chimeras were detected and removed with UCHIME [38], and clustering was performed using SWARM [39] in the FROGS pipeline. The minimum sequence abundance proportion was set at 5e^− 5^ to keep OTUs with a minimum prevalence fixed at 4. Taxonomic assignments of 16S rRNA, ITS, and 28S rRNA-based OTUs were performed using Silva138.1 [40], Unite8.2 [41], and MaarjAM (28S) [42], respectively. For the 16S rRNA gene of bacteria, the sequences “multi-affiliated” at the phylum level were removed, as well as those affiliated to grapevine mitochondrial and chloroplastic16S rRNA sequences. As the proportions of host 16S rRNA sequences were important in the root endosphere, the samples from root and rhizosphere were divided before rarefaction at the number of sequences in the sample containing the fewest ones. Only the samples containing more than 4,000 or 250 sequences were kept for the rhizosphere and the root endosphere, respectively. For the fungal ITS region, the sequences “multi-affiliated” or “unidentified” at the phylum level were removed and only the samples containing more than 4,000 sequences were kept. For the 28S rRNA gene of AMF, the sequences affiliated to “New_clade” at the class level were removed and only the samples containing more than 5,000 sequences were kept.

### Metabarcoding data processing and prediction of microbial functions

Bacterial functions and pathways were predicted using phylogenetic investigation of communities by reconstruction of unobserved states (PICRUSt2) [43]. The tool was run on the FROGS pipeline installed in a Galaxy instance [34]. EPA-ng was used to construct the reference tree with a minimum alignment length of 0.8. Bacterial functions abundance was predicted using KO database with a NSTI cut-off set at 0.5, and an identity and coverage alignment cut-off at 0.9. Obtained abundances of pathways were measured using the KEGG database and the final output tables were normalized (values were divided by the sum of columns, then multiplied by 10^6^). Only the “Metabolism” and “Environmental Information Processing” classifications were kept for statistical analyses. Fungal trophic modes and guilds were predicted at the genus level using the “funguild_assign” function from the *FUNGuildR* (0.2.0.9000) package and the FUNGuild database [44]. Only the confidence ranking “probable” and “highly probable” were selected for statistical analyses. OTUs assigned to more than two trophic modes and more than two guilds were classified as “multi-affiliated”. The OTUs “unassigned” were removed before comparisons were made.

### Statistical analyses

All the statistical analyses were performed on R (v4.2.1) using RStudio (2022.07.1). Figures were generated with *ggplot2* (v3.4.1) and *ggthemes* (v4.0.4) and arranged with *ggpubr* (v0.4.0).

Chao1 and Simpson indexes were calculated after data filtration and rarefaction, using the “estimate_richness” function and PCoA based on the Bray-Curtis distance were performed using the “plot_ordination” function, both from *phyloseq* (1.38.0) [45]. Linear Discriminant Analysis Effect Size (LEfSe) was carried out using the “run_lefse” function from *microbiomeMarker* (1.2.2) with data transformation (log10), lda_cutoff fixed at 4, kw_cutoff and wilcoxon_cutoff at 0.05 [46]. For comparisons of α-diversity metrics between the root system compartments, student t-tests or Wilcoxon tests were performed after checking assumptions for parametric tests with Shapiro (normality) and Bartlett (homogeneity of variance) tests, respectively. Two-way Analysis of Variance (ANOVA) with the genotype and block factors were performed on α-diversity metrics, bacterial predicted pathways, and results obtained with cultivable and qPCR approaches. The genotype-block interaction was tested prior to this test. Assumptions for parametric tests were checked on the residuals with Shapiro (normality) and Bartlett (homoscedasticity) tests. Comparison between genotypes were carried out with “pairwise.t.test” or “pairwise.wilcox.test” functions with Bonferroni correction from the *stats* package (4.2.1). To test the effects of the genotype and block on the Bray-Curtis index, Permutational Analysis of Variance (PERMANOVA) with 999 permutations were performed with the “adonis2” function from *vegan* package (2.6.4). Genotypes were then compared together using the “pairwise.adonis2” function from the *pairwiseAdonis* package (0.4.1). Heatmaps were generated using Euclidian distance to compare the abundances of bacterial metabolic pathways between genotypes using the “pheatmap” function from *pheatmap* (1.0.12). The effect of genotypes on the proportion of trophic modes and guilds were assessed with a chi-squared test using “chisq.test” function from *stats* package. PCA were assessed with “PCA” function from *FactoMineR* (2.8) and plotted using “fviz_pca_ind” function from *factoextra* (1.0.7) to compare predicted bacterial functions between genotypes. Matrices showing only the significant correlations were established using the ggcorrplot function from *ggcorplot* (0.1.4) with the “square” method and *P*-values were calculated using the “cor.mtest” function.

## Results

### Rhizosphere and root endosphere present distinct microbial communities

**Fig. 1:**
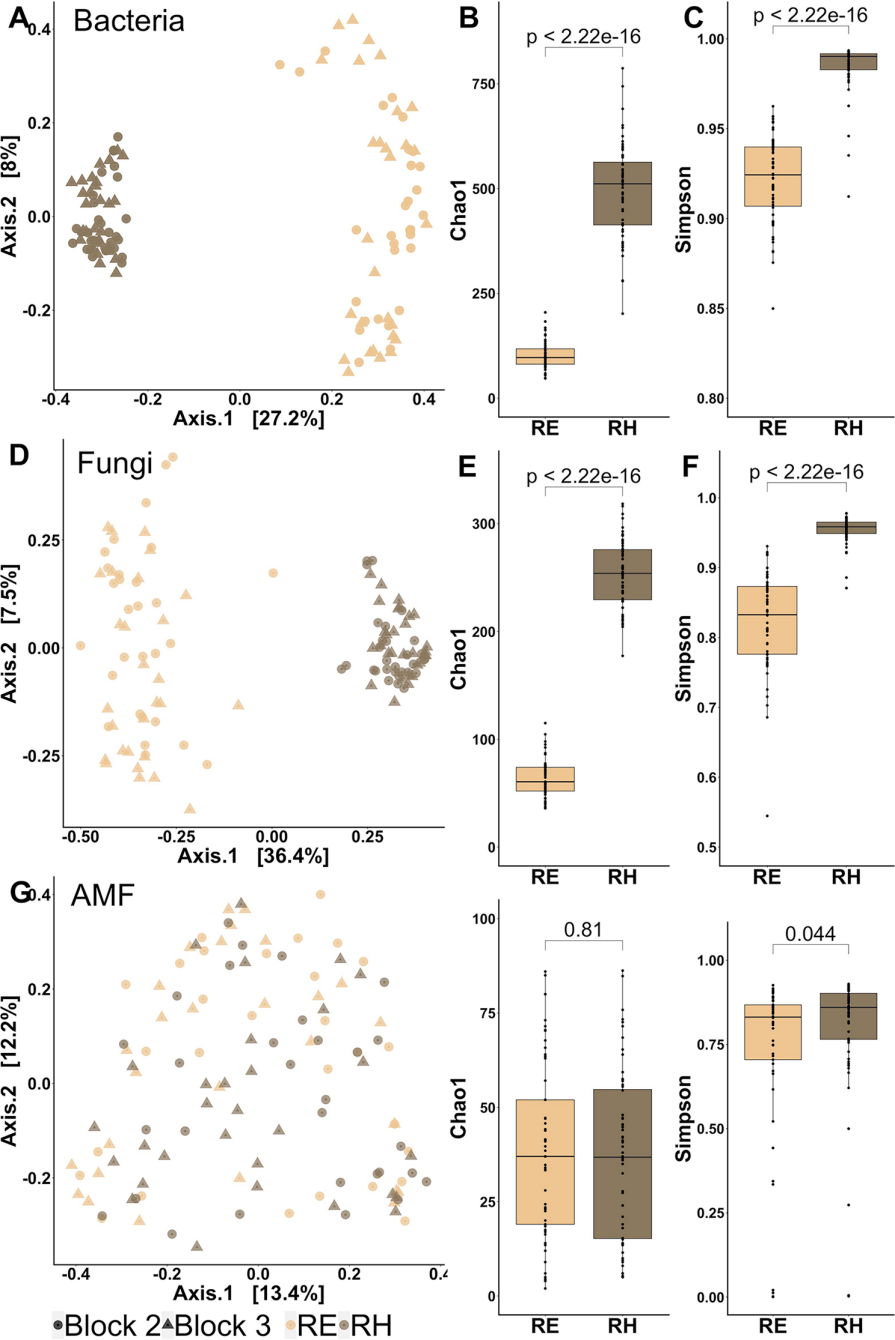
Comparison of microbial communities between the root endosphere (RE, light brown) and the rhizosphere (RH, dark brown). Comparison of β-diversity by principal coordinate analysis (PCoA) of (**A**) bacterial, (**D**) fungal and AMF (**G**) communities based on Bray–Curtis dissimilarity matrices. Comparison of α-diversity with richness (Chao1) and diversity (Simpson) of bacterial (**B, C**), fungal (**E, F**) and AMF communities (**H, I**). *P*-values were calculated using Student or Wilcoxon tests and were considered as significant when < 0.05 (*n* = 60)

In the root system (rhizosphere and root endosphere), 1450 bacterial OTUs were successfully affiliated to 16 phyla, 41 classes, 85 orders, 115 families, 167 genera and 70 species. The bacterial community associated to the globality of the root system was composed of *Proteobacteria* (43%), *Actinobacteriota* (32%), *Acidobacteriota* (7%), *Chloroflexi* (5%), *Bacteroidota* (3%), *Verrucomicrobiota* (3%), *Firmicutes* (2%), *Myxococcota* (2%), *Planctomycetota* (2%) and other phyla whose proportions were less than 1% (*Dependentiae*, *Desulfobacterota*, *Gemmatimonadota*, *Latescibacterota*, *Methylomirabilota*, *Nitrospirota* and RCP2-54) (Additional file 1: Fig. S1A). PCoA displayed a strong clustering of the bacterial communities according to the compartments and were primarily differentiated along the first axis (Fig. 1A). A higher dispersion of the individuals was observed in the root endophytes group along the second axis. Bacterial Chao1 (Fig. 1B) and Simpson (Fig. 1C) indexes were significantly higher in the rhizosphere than in the root endosphere. Bacterial OTUs were primarily exclusives (66%) of the rhizosphere, while 31% were common between the root system compartments and less than 3% were root exclusives (Additional file 1: Fig. 2A). Root system compartments displayed bacterial dissimilarities at the phylum (Additional file 1: Fig. 1A) and the class (Additional file 1: Fig. 1B) levels. The LEfSe detected an enrichment of 7 phyla (*Acidobacteriota*, *Chloroflexi*, *Verrucomicrobiota, Planctomycetota, Firmicutes, Actinobacteriota, Gemmatimonadota*), 10 classes, 16 orders, 17 families, 14 genera and 13 species in the rhizosphere, compared to 3 phyla (*Proteobacteria, Bacteroidota, Dependentiae)*, 5 classes, 8 orders, 14 families, 17 genera, 16 species in the root endosphere (Additional file 1: Fig. 3A).

Among the fungal community analyzed by ITS sequencing, 650 OTUs were affiliated to 12 phyla, 31 classes, 69 orders, 115 families, 174 genera and 302 species. The root system community was composed of *Ascomycota* (60%), *Basidiomycota* (22%), *Rozellomycota* (11%), *Mortierellomycota* (5%), *Glomeromycota* (2%) and other phyla whose proportions were less than 1% (*Blastocladiomycota*, *Calcarisporiellomycota*, *Chytridiomycota*, *Kickxellomycota*, *Mucoromycota*, *Olpidiomycota*, *Zoopagomycota*) (Additional file 1: Fig. S1C). PCoA enabled separation of the fungal community into two groups corresponding to the compartments of the root system, with the same profile as that observed for bacteria (Fig. 1D). Fungal Chao1 (Fig. 1E) and Simpson (Fig. 1F) indexes were significantly higher in the rhizosphere than in the root endosphere. Amongst the root system compartments, 62% of the fungal OTUs were shared, with 38% and less than 1% specific to the rhizosphere and the root endosphere, respectively (Additional file 1: Fig. S2A). Dissimilarities were observed at the phyla (Additional file 1: Fig. S1C) and the class (Additional file 1: Fig. S1D) levels between the root system compartments. LEfSe showed an enrichment of 2 phyla (*Ascomycota, Mortierellomycota)*, 5 classes, 9 orders, 12 families, 12 genera and 10 species in the rhizosphere. In the root endosphere, 2 phyla (*Glomeromycota, Basidiomycota)*, 5 classes, 7 orders, 9 families, 12 genera and 14 species were enriched (Additional file 1: Fig. S3B).

In the fungal sub-group of AMF, 230 OTUs were affiliated to 1 phylum, 2 classes, 3 orders, 5 families, 8 genera, and 40 species. The AMF genera were represented by *Glomus* (86%), *Claroideoglomus* (3%), *Paraglomus* (2%), *Scutellospora* (2%), multi-affiliated genera (6%), and other genera whose proportions were less than 1% (*Acaulospora*, *Rhizophagus*, and *Septoglomus*) (Additional file 1: Fig. S1E). No segregation of the root system compartments was observed with the PCoA (Fig. 1G). Although no significant difference was observed for the Chao1 index (Fig. 1H) between root system compartments, the Simpson index (Fig. 1I) was significantly higher in the rhizosphere than in the roots. Amongst both root and rhizosphere compartments, 88% of AMF OTUs were shared, with 11% and less than 1% specific to the rhizosphere and the root endosphere, respectively (Additional file 1: Fig. S2A). The abundance graph at the genus level showed different proportions of AMF between root endosphere and rhizosphere compartments (Additional file 1: Fig. S1E). LEfSe revealed an enrichment of 1 order (*Paraglomerales*), 2 families (*Paraglomeraceae* and *Claroideoglomeraceae*), 2 genera and 2 species in the rhizosphere, compared to 1 order (*Glomerales*) and 1 family (*Glomeraceae*) in the root endosphere (Additional file 1: Fig. S3C). Although the differences amongst the root system compartments were less evident for the AMF, we decided to separate the root endosphere and the rhizosphere in the following analyses for the three groups of microorganisms.

### Rootstock and scion genotypes influence the bacterial community of the rhizosphere and the root endosphere

**Table 1:** Influence of the rootstock and the scion genotypes on the α-diversity (richness = Chao1, diversity = Simpson) and the β-diversity metrics (Bray-Curtis dissimilarity) amongst bacteria, fungi and AMF, in the rhizosphere (RH) and the root endosphere (RE). *P*-values were calculated using two-way Anova and were considered as significant when the *p*-value < 0.05, PVE = percentage of variance explained

			Influence of the rootstock									Influence of the scion								
			Chao1			Simpson			Bray-Curtis			Chao1			Simpson			Bray-Curtis		
			p-value		PVE	p-value		PVE	p-value		PVE	p-value		PVE	p-value		PVE	p-value		PVE
**Bacteria**	**RH**	**Genotype (G)**	**1.69e-08**	***	**73%**	**2.99e-4**	***	**53%**	**0.001**	***	**50%**	0,574	ns	/	**0.008**	**	**41%**	**0.002**	**	**25%**
		**Block (B)**	**0.012**	*	**5%**	0.485	ns	/	**0.003**	**	**7%**	**0.001**	***	**36%**	0.408	ns	/	**0.019**	*	**6%**
		**G x B**	**1.85e-05**	***	**15%**	0.919	ns	/	**0.001**	***	**17%**	**0.031**	*	**22%**	0.749	ns	/	**0.001**	***	**33%**
	**RE**	**G**	0.663	ns	/	**0.018**	*	**29%**	**0.001**	***	**42%**	0.469	ns	/	0.632	ns	/	**0.004**	**	**24%**
		**B**	0.826	ns	/	**0.002**	**	**19%**	**0.019**	*	**4%**	0.861	ns	/	0.483	ns	/	**0.044**	*	**6%**
		**G x B**	0.644	ns	/	0.512	ns	/	**0.045**	*	**12%**	0.476	ns	/	0.318	ns	/	**0.001**	***	**25%**
**Fungi**	**RH**	**G**	0.141	ns	/	**0.007**	**	**40%**	**0.001**	***	**42%**	0.990	ns	ns	0.232	ns	/	**0.001**	***	**30%**
		**B**	**0.008**	**	**18%**	0.590	ns	/	**0.002**	**	**6%**	0.193	ns	ns	0.754	ns	/	**0.001**	***	**7%**
		**G x B**	**7.67e-06**	***	**45%**	0.168	ns	/	**0.001**	***	**19%**	**1.79e-4**	***	**60%**	**0.045**	*	**30%**	**0.001**	***	**29%**
	**RE**	**G**	**0.001**	**	**49%**	0.052	ns	/	**0.001**	***	**33%**	0.490	ns	/	0.727	ns	/	**0.002**	**	**24%**
		**B**	0.897	ns	/	0.389	ns	/	**0.008**	**	**5%**	0.558	ns	/	0.318	ns	/	**0.015**	*	**7%**
		**G x B**	0.729	ns	/	0.694	ns	/	**0.001**	***	**19%**	0.279	ns	/	0.136	ns	/	**0.001**	***	**26%**
**AMF**	**RH**	**G**	0.395	ns	/	0.239	ns	/	**0.001**	***	**23%**	0.409	ns	/	0.471	ns	/	**0.001**	***	**22%**
		**B**	0.373	ns	/	**0.033**	*	**12%**	**0.042**	*	**4%**	0.284	ns	/	0.489	ns	/	0.438	ns	/
		**G x B**	0.194	ns	/	0.056	ns	/	**0.006**	**	**17%**	0.374	ns	/	0.284	ns	/	**0.031**	*	**16%**
	**RE**	**G**	**0.007**	**	**41%**	**0.013**	*	**37%**	**0.031**	*	**19%**	0.191	ns	/	0.516	ns	/	0.137	ns	/
		**B**	0.385	ns	/	0.210	ns	/	0.523	ns	/	0.997	ns	/	0.174	ns	/	0.299	ns	/
		**G x B**	0.131	ns	/	0.233	ns	/	0.278	ns	/	0.548	ns	/	0.098	ns	/	**0.002**	**	**24%**

**Fig. 2:**
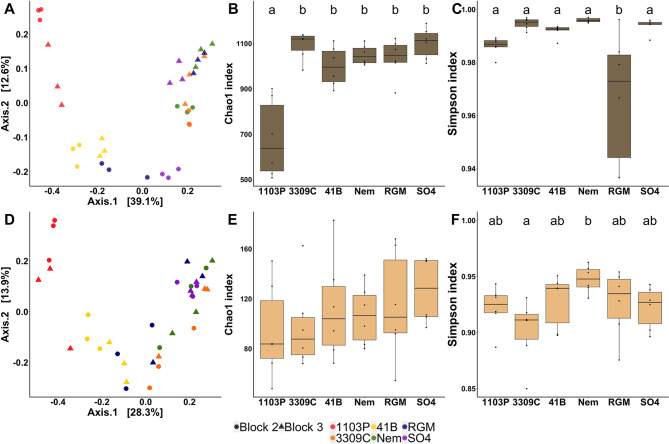
Comparison of bacterial communities in the rhizosphere (dark brown, top panel) and the root endosphere (light brown, bottom panel) between the 6 rootstock genotypes grafted onto CS (*n* = 6). Comparison of bacterial communities by principal coordinate analysis (PCoA) based on Bray–Curtis dissimilarity matrices in the rhizosphere (**A)** and the root endosphere (**D)**. Comparison of bacterial richness (Chao1) and diversity (Simpson) in the rhizosphere (**B**, **C**) and the root endosphere (**E**, **F**). *P*-values were calculated using Pairwise-Student tests with Bonferroni correction and were considered as significant when the adjusted *p*-value < 0.05

**Fig. 3:**
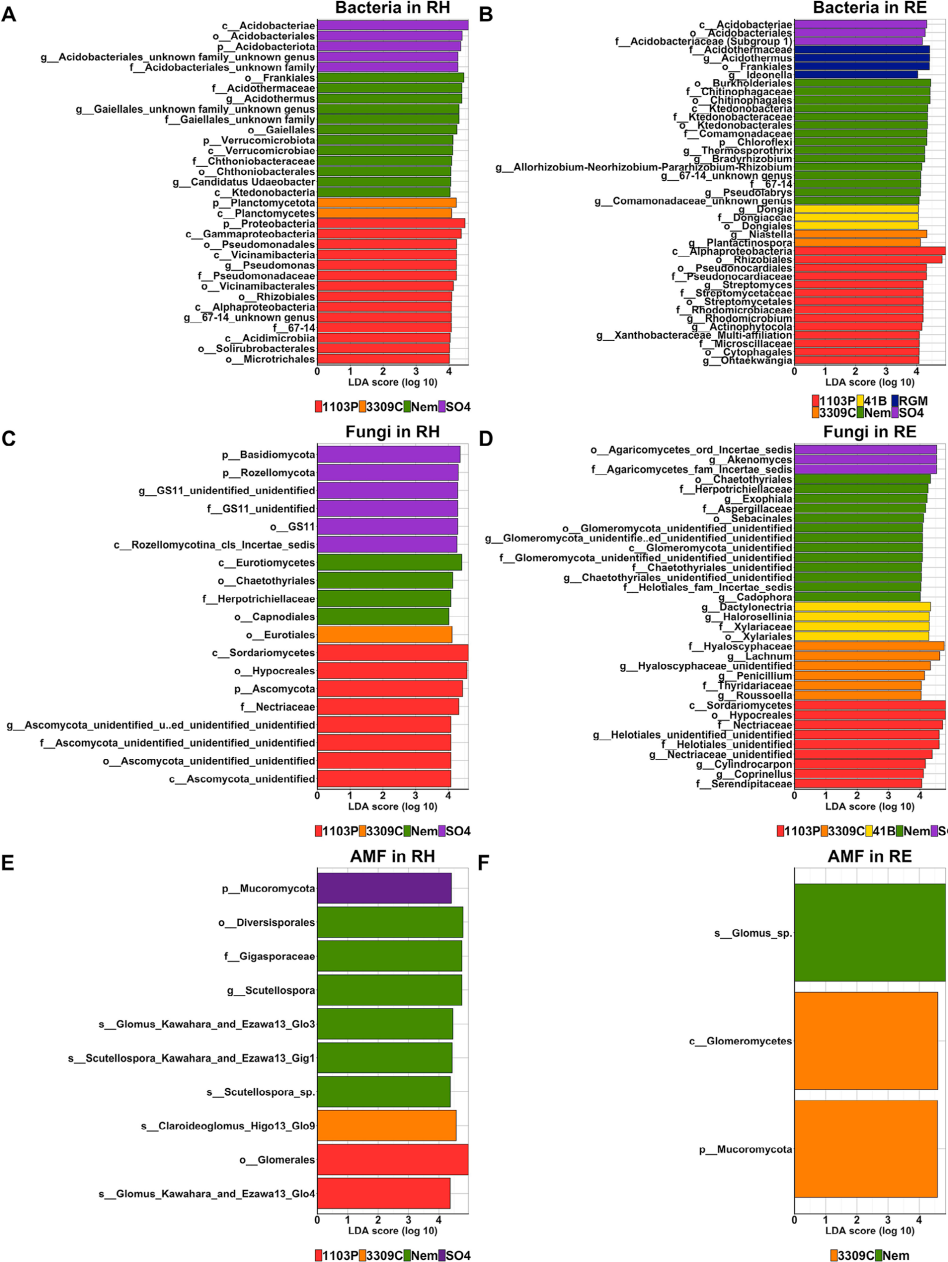
Histogram of the linear discriminant analysis (LDA) scores reveals the most differentially abundant taxa of bacteria (**A**, **B**), fungi (**C**, **D**) and AMF (**D**, **E**) in the rhizosphere (RH) and the root endosphere (RE) between rootstock genotypes grafted with CS.

When the bacterial communities of the six rootstocks grafted with CS were assessed, no effect of the rootstock genotype was observed on rhizosphere bacteria level with both cultivable and qPCR methodologies (Additional file 2: Table S4; Additional file 1: Fig. S4A). The rootstock genotype, the block, and the factor combinations significantly influenced the bacterial Bray-Curtis index in the rhizosphere compartment with a percentage of variance explained (PVE) of 50, 7, and 17%, respectively (Table 1). In the root endosphere, similar results were obtained with 42, 4, and 12% of PVE, respectively. The Chao1 and Simpson indexes were influenced by the rootstock genotype (73 and 53% of PVE respectively) in the rhizosphere. The Chao1 index was also influenced by the block (5% of PVE) and the factor interactions (15% of PVE). In the root endosphere, only the Simpson index was influenced by the genotype and the block (29 and 19% of PVE respectively). As the effect of the genotype (expressed as the percentage of variance explained) was always stronger than that of the block or the factor interactions, we kept the genotype factor to compare rootstocks together with a suitable number of biological replicates (*n* = 6). PCoA showed a genotype-dependent clustering of individuals in both rhizosphere (Fig. 2A) and root endosphere (Fig. 2D) compartments. Interestingly, the Bray-Curtis index measured for 1103P rootstock was significantly different from those of all other genotypes in both root system compartments, except for SO4 rootstock in the root endosphere (Additional file 2: Table S5). Moreover, the Bray-Curtis index of 41B rootstock was significantly different from those of Nemadex and SO4 in the rhizosphere, as well as those of 3309 C in both root system compartments. In the rhizosphere, 1103P rootstock had a significantly lower Chao1 index (Fig. 2B) than all the other genotypes, while RGM had the significantly lowest Simpson index (Fig. 2C). In the root endosphere, no significant differences were detected between bacterial richness amongst the rootstock (Fig. 2E), while the Simpson index of 3309 C rootstock was significantly lower compared to Nem (Fig. 2F). Between the six different rootstock genotypes, 47% of the rhizospheric OTUs were common, while less than 1% were specific from one genotype (Additional file 1: Fig. S2B). Inversely in the root endosphere, only 6% of bacterial OTUs were common and 49% were genotype-exclusives. LEfSe detected an enrichment of 4 phyla (*Acidobacteriota* for SO4, *Verrucomicrobiota* for Nemadex, *Planctomycetota* for 3309 C and *Proteobacteria* for 1103P- rootstocks), 7 classes, 7 orders, 6 families, 6 genus, 6 species in the rhizosphere amongst the six rootstocks (Fig. 3A). In the root endosphere, 3 classes, 10 orders, 11 families, 16 genus and 17 species were enriched (Fig. 3B).

Regarding the influence on microbial communities of five scion genotypes grafted onto RGM rootstock, an effect of the scion on the level of bacterial 16S rRNA gene from the rhizosphere was reported (Additional file 2: Table S4), but no significant difference was observed between groups with the adjusted *P*-values (Additional file 2: Fig. S4D). In the rhizosphere and the root endosphere, Bray-Curtis index was influenced by the scion genotype (25 and 24% of PVE), the block (6% of PVE) and their interaction (33 and 25% of PVE) (Table 1). Regarding the bacterial α-diversity, the effect of the scion genotype was only reported on the Simpson index in the rhizosphere, explaining 41% of PVE. Individuals were not well clustered by scion genotype according to PCoA plot for both rhizosphere (Additional file 1: Fig. S5A) and root endosphere (Additional file 1: Fig. S5D) compartments. Only the Bray-Curtis index of UB scion was significantly different from the one of Syrah grafted onto RGM in both root system compartments, as well as the Bray-Curtis indexes of UB and Grenache in the rhizosphere (Additional file 2: Table S5). Simpson index in the rhizosphere was significantly lower in CS than in Grenache and UB (Additional file 1: Fig. S5C). Bacterial OTUs were common between scion genotypes at 78% and less than 1% were genotype exclusive in the rhizosphere, compared to 18% and 40% in the root endosphere (Additional file 1: Fig. S2C). The LEfSe identified genotype-dependent enrichments of 2 phyla (*Planctomycetota* for UB, *Proteobacteria* for Grenache), 2 classes, 1 order, 1 family, 1 genus and 1 species in the rhizosphere (Additional file 1: Fig. S6A), compared to 2 phyla (*Acidobacteriota* for UB and *Proteobacteria* for Grenache) 2 orders, 2 families, 5 genera and 6 species in the root endosphere (Additional file 1: Fig. S6B).

### Both genotypes of the grafted plant influence the root-associated fungal community

**Fig. 4:**
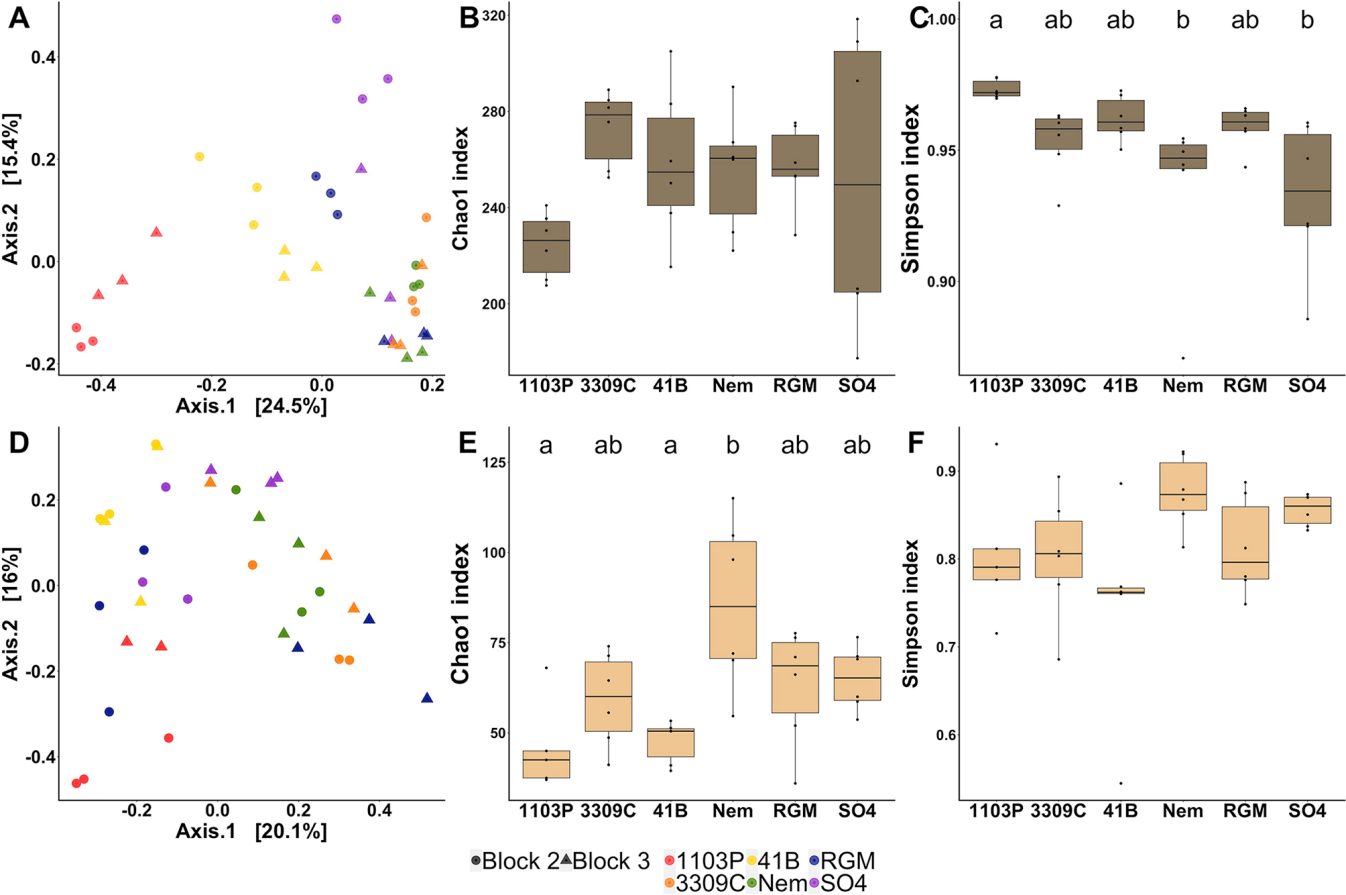
Comparison of fungal communities in the rhizosphere (top panel, dark brown) and the root endosphere (bottom panel, light brown) between the 6 rootstock genotypes grafted with CS (*n* = 6). Comparison of fungal communities by principal coordinate analysis (PCoA) based on Bray–Curtis dissimilarity matrices in the rhizosphere (**A)** and the root endosphere (**D)**. Comparison of fungal richness (Chao1) and diversity (Simpson) in the rhizosphere (**B**, **C**) and the root endosphere (**E**, **F**). *P*-values were calculated using Pairwise-Student tests with Bonferroni correction and were considered as significant when the adjusted *p*-value < 0.05

Quantitative PCR analysis revealed that the rootstock genotype affected the level of the fungal ITS gene from the rhizosphere (Additional file 2: Table S4) which was significantly higher for SO4 rootstock than for 1103P, 41B and RGM (Additional file 1: Fig. S4B). In the rhizosphere and the root endosphere, the Bray-Curtis index of fungal communities was influenced by the rootstock genotype (explaining 42% and 33% of PVE), the block (6% and 5% of PVE) and their interaction (19% of PVE) (Table 1). Regarding α-diversity metrics, the Simpson index in the rhizosphere and the Chao1 index in the root endosphere were influenced by the rootstock genotype only (40 and 49% of PVE, respectively). PCoA displayed strong genotype-dependent clustering in the rhizosphere (Fig. 4A). The fungal Bray-Curtis index of 1103P rootstock was significantly different from those of all other genotypes except for 41B (Additional file 2: Table S6). Other distinct fungal Bray-Curtis indexes were observed between Nemadex and 41B, as well as 3309 C and RGM or 41B. The clustering of fungal communities according to rootstock genotype was less evident in the root endosphere (Fig. 4D). The fungal structure was significantly different between 1103P rootstock and Nemadex, 41B, and 3309 C, as well as between 41B rootstock and Nemadex and 3309 C (Additional file 2: Table S6). In the rhizosphere, the Simpson index was significantly higher in 1103P rootstock than in Nemadex and SO4 (Fig. 4C). In the root endosphere, the Chao1 index was significantly higher in Nemadex rootstock than in 1103P and 41B (Fig. 4E). In the rhizosphere, fungal OTUs were common and genotype-exclusive at 32% and 10%, respectively, while 7% were common and 34% were genotype-exclusive in the root endosphere (Additional file 1: Fig. S2B). Three phyla (*Ascomycota* for 1103P, *Basidiomycota* and *Rozellomycota* for SO4), 4 classes, 6 orders, 4 families, 2 genera, 2 species were enriched in the rhizosphere of rootstock genotypes (Fig. 3C). In the root endosphere, 2 classes, 7 orders, 14 families, 17 genera and 19 species were enriched (Fig. 3D).

Despite the reported effect of the scion on the level of fungal 18S rRNA gene from the rhizosphere (Additional file 2: Table S4), no significant differences were observed between scion genotypes using the adjusted *P*-values (Additional file 1: Fig. S4E). In the rhizosphere and the root endosphere, the fungal Bray-Curtis index was influenced by the scion genotype (30% and 24% of PVE), the block (7% of PVE) and their interaction (29% and 26% of PVE) (Table 1). None of the fungal α-diversity metrics were influenced by the scion genotype or the block. However, effects of their interaction were reported on the richness and the diversity of the rhizosphere (60% and 30% of PVE, respectively) (Table 1). According to PCoA, individuals were not clustered by genotypes in both rhizosphere (Additional file 1: Fig. S7A) and root endosphere (Additional file 1: Fig. S7D) compartments. The fungal Bray-Curtis index measured for UB was significantly different from those of PN, Syr and Gre in the rhizosphere, and from those of PN in the root endosphere (Additional file 2: Table S6). Syr and Gre were also significantly different in the rhizosphere. In this compartment, 40% of fungal OTUs were common and 11% were genotype-exclusive, while 17% were common and 33% were genotype-exclusive in the root endosphere. LEfSe analysis detected enrichments in 3 scion genotypes of 2 classes, 3 orders, 2 families, 2 genera and 2 species in the rhizosphere (Additional file 1: Fig. S6C), while enrichments of 3 classes, 2 orders, 2 families, 2 genera and 2 species were detected in the root endosphere (Additional file 1: Fig. S6D).

### Rootstock genotypes drive the AMF community in the root endosphere

**Fig. 5:**
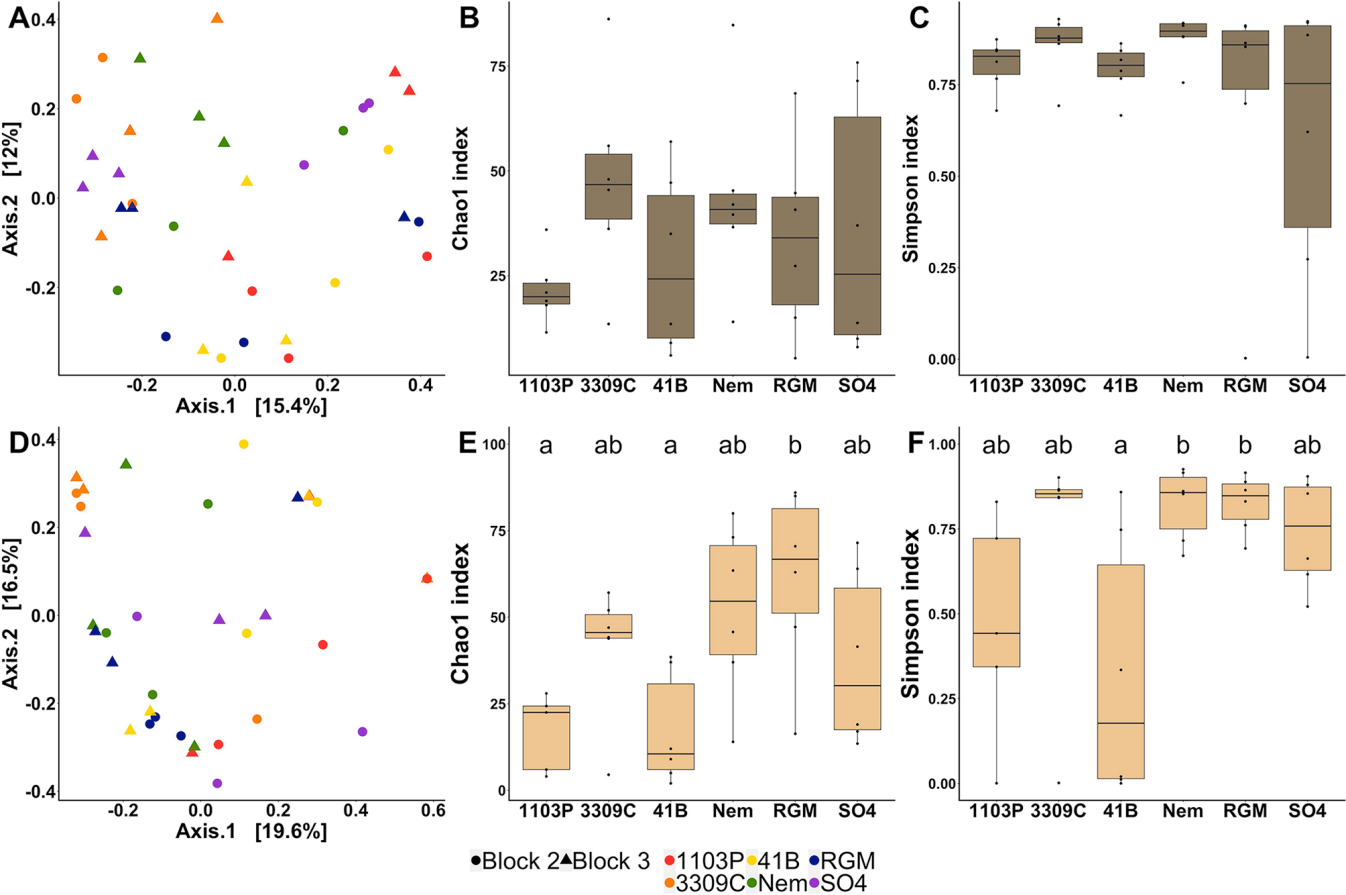
Comparison of AMF communities in the rhizosphere (top panel, dark brown) and the root endosphere (bottom panel, light brown) between the 6 rootstock genotypes grafted with CS (*n* = 6). Comparison of AMF communities by principal coordinate analysis (PCoA) based on Bray–Curtis dissimilarity matrices in the rhizosphere (**A)** and the root endosphere (**D)**. Comparison of AMF richness (Chao1) and diversity (Simpson) in the rhizosphere (**B**, **C**) and the root endosphere (**E**, **F**). *P*-values were calculated using Pairwise-Student tests with Bonferroni correction and were considered as significant when the adjusted *p*-value < 0.05

The Bray-Curtis index measured for AMF was influenced by the rootstock genotype, the block, and the interaction in the rhizosphere (23%, 4%, and 17% of PVE, respectively), as well as by the genotype in the root endosphere (19% of PVE) (Table 1). However, Chao1 and Simpson indexes were influenced by the genotype only in the root endosphere (41% and 37% of PVE, respectively). PCoA failed to cluster individuals according to rootstock genotypes in both rhizosphere (Fig. 5A) and root endosphere (Fig. 5D) compartments. Only the Bray-Curtis index of 3309 C rootstock was significantly distinct from the indexes of 1103P and 41B in the rhizosphere (Additional file 2: Table S7). In the root endosphere, the Chao1 index was significantly higher in RGM than in 1103P and 41B (Fig. 5E). Likewise, the Simpson index was higher in RGM and Nemadex than in 41B (Fig. 5F). In the rhizosphere, 12% of the AMF OTUs were common between the rootstocks while 26% were genotype exclusive (Additional file 1: Fig. S2B). In the root endosphere, 20% were common and 30% were exclusive. LEfSe detected an enrichment of 1 phylum, 2 orders, 1 family, 1 genus and 5 species in the rhizosphere (Fig. 3F), compared to 1 phylum, 1 class and 1 species in the root endosphere (Fig. 3E).

In addition to rootstock, a scion genotype effect was reported on AMF communities, the Bray-Curtis index was influenced by both the genotype and its interaction with the block in the rhizosphere (22 and 16% of the PVE, respectively), as well as by the factor interactions in the root endosphere (24% of PVE) (Table 1). However, the α-diversity indices did not detect any effects of the scion genotype in either of the root system compartments. According to PCoA, individuals were not clustered by genotype in either the rhizosphere (Additional file 1: Fig. S8A) or the root endosphere (Additional file 1: Fig. S8D) compartments. No AMF communities were significantly distinct between the scion genotypes with the Bray-Curtis index in either of the root system compartments endosphere (Additional file 2: Table S7). In the rhizosphere, 14% and 27% of the AMF OTUs were common or exclusive between scion genotypes, respectively, while in the root endosphere, 20% were common and 28% were genotype exclusive (Additional file 1: Fig. S2C). LEfSe detected an enrichment of 1 phylum, 1 genus and 4 species in the rhizosphere (Additional file 1: Fig. S6E), compared to 1 phylum, 1 class and 2 species in the root endosphere (Additional file 1: Fig. S6F).

### Predicted functions of the bacterial and fungal microbiomes and impact of the root system microbiomes on plant phenotypic traits

**Fig. 6:**
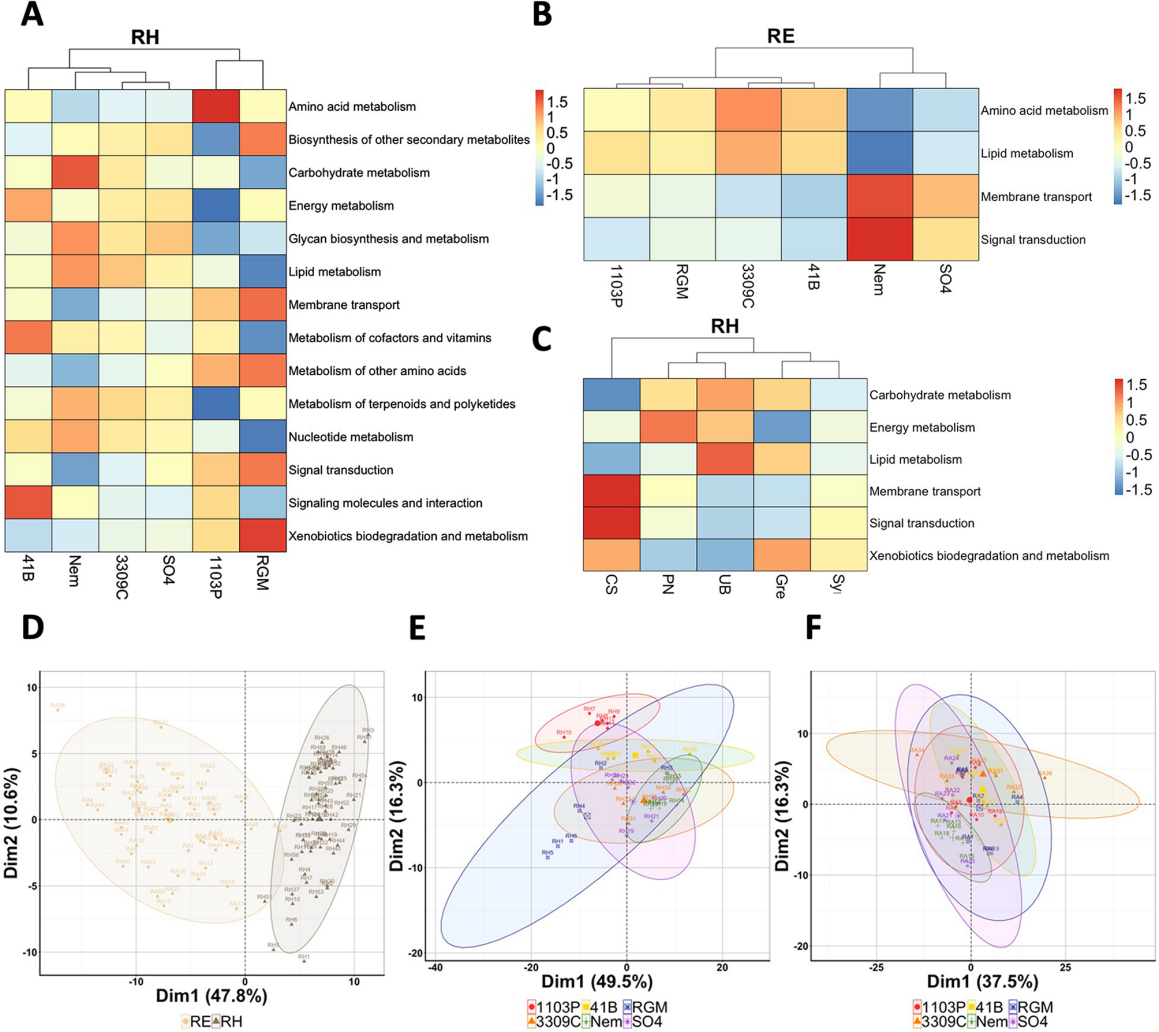
PICRUSt inference of bacterial metabolic pathways and functions. Heatmap showing the abundance of bacterial metabolic pathways which were significantly influenced by the rootstock genotype in (**A**) the rhizosphere and (**B**) the root endosphere, and by (**C**) the scion genotypes in the rhizosphere (*n* = 6). PCA to compare the predicted functions of bacteria (119 variables) between (**D**) roots system compartments (*n* = 60), and rootstock genotypes in (**E**) the rhizosphere and (**F**) the root endosphere (*n* = 6)

Bacterial potential metabolic pathways were predicted using PICRUSt2. All the abundances of pathways were significantly higher in the rhizosphere than in the root endosphere, except for the metabolism of lipids, the metabolism of other amino acids, and the xenobiotic biodegradation and metabolism (Additional file 2: Table S8). PCA performed with the 119 predicted functions showed high clustering dependent on the root system compartment (Fig. 6D), suggesting that the function of the bacterial microbiomes depends on their environment. An effect of the rootstock genotype was reported on the abundances of all the predicted bacterial metabolic pathways in the rhizosphere (Additional file 2: Table S9). Several biosynthetic pathways were very abundant in the rhizosphere of certain rootstock genotypes, such as the amino acid metabolism for 1103P, the carbohydrate metabolism for Nemadex, the signaling molecules interaction for 41B, and the xenobiotics biodegradation and metabolism for RGM (Fig. 6A). According to PCAs, the predicted functions of the rhizospheric bacterial community for 1103P rootstock were distinct from those of 3309 C and Nemadex (Fig. 6E). In the root endosphere, the rootstock genotype influenced only the metabolisms of amino acids and lipids, the membrane transport, and the signal transduction of bacteria (Additional file 2: Table S9; Additional file 1: Fig. S6B). However, according to PCA, a slight segregation was detected between Nemadex and 41B (Fig. 6F). The abundances of six predicted bacterial metabolic pathways in the rhizosphere were influenced by the scion genotype (metabolism of carbohydrate, energy and lipid, membrane transport, signal transduction and xenobiotics biodegradation and metabolism), while the abundances of three and four pathways were influenced by the block and the factors combination, respectively (Additional file 2: Table S9; Fig. 6C). According to PCA, no scion genotype-dependent clustering of bacterial functions was observed in either the rhizosphere (Additional file 1: Fig. S9A) or root endosphere (Additional file 1: Fig. S9B) compartments. Here, the influence of the scion on the abundances of bacterial metabolic pathways is weaker than that of the rootstock.

**Fig. 7:**
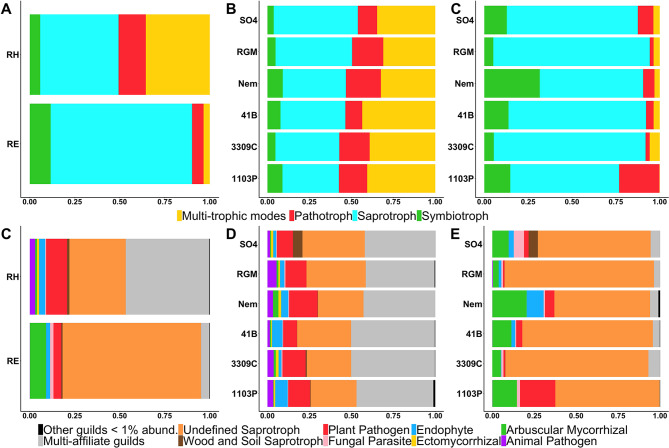
Comparisons of the predicted trophic modes and guilds between the root system compartments (**A**, **C**) or the rootstock genotypes in the rhizosphere (**B**, **D**) and the root endosphere (**C**, **E**)

Fungal trophic modes and guilds, predicted using the FUNGuild database, were successfully affiliated with 33% of fungal OTUs from the rhizosphere and 42% from the root endosphere. An effect of the root system compartments on the proportions of fungal trophic modes (Fig. 7A) and guilds (Fig. 7C) was reported (Chi Squared test: *P*-values < 2.2e-16). Fungi were mainly saprotroph in both root system compartments, even though a significant number of multi-trophic modes were observed in the rhizosphere. The highest proportions of pathotroph and plant pathogen were observed in the rhizosphere, whereas the highest proportions of symbiotroph or arbuscular mycorrhizal were observed in the roots. Rootstock genotype influenced the proportions of fungal trophic modes (Fig. 7B) and guilds (Fig. 7D) in both root system compartments (Chi Squared test: *P*-values < 2.2e-16). Interestingly, 1103P had over 3 times more pathogenic fungi than any other genotype in the root endosphere. Moreover, the highest proportion of AMF in roots was observed for Nemadex rootstock. An effect of the scion was also detected on the proportions of trophic modes and guilds in both root system compartments (Chi Squared test: *P*-values < 2.2e-16, Additional file 1: Fig. S10). The root system of RGM grafted with UB had over twice as much AMF in the roots than other scion genotypes.

**Fig. 8:**
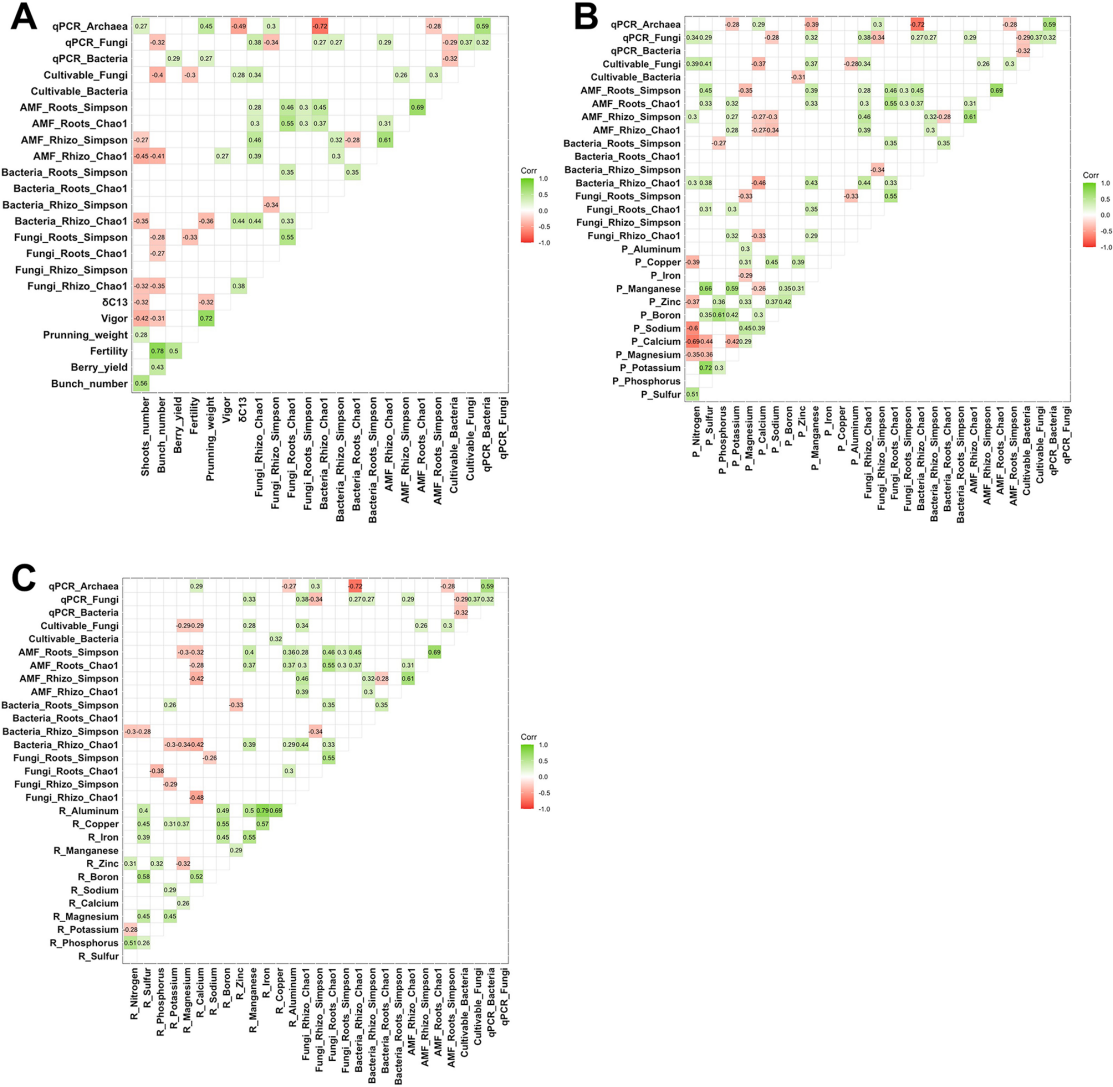
Matrices of correlations established between the variables measured to characterize the root system microbiomes and the plant phenotype (**A**), the mineral status of petioles (**B**) and the mineral status of roots (**C**). All colored boxes correspond to significant correlations (*p*-value < 0.05)

To study the impacts of microbial communities on plant phenotypic traits, and mineral content in the roots and petioles, correlation matrices were established with the microbial variables (cultivable, qPCR and metabarcoding approaches). Between the root system microbiomes and the plant phenotypic traits, 22 significant correlations were established, with r values ranging from − 0.49 to 0.45. The strongest correlations (*P*-values < 0.05) were obtained between the number of shoots and AMF richness in the rhizosphere (*r* = -0.45); the number of bunches and the cultivable fungi (*r* = -0.4) and the AMF richness in the rhizosphere (*r* = 0.41); the bunch pruning weight and the rhizosphere archaeal level measured by qPCR (*r* = 0.45); the δ^13^C and the rhizosphere archaeal level (*r* = -0.49) and the bacterial richness in the rhizosphere (*r* = 0.44) (Fig. 8A). In addition, 39 microbial variables were significantly correlated to mineral dosage in the petiole, with r values ranging from − 0.44 to 0.45. The strongest correlations were obtained between the sulfur content and the AMF diversity in the roots (*r* = 0.45); the richness of rhizosphere bacteria and the calcium content (*r* = -0.46) and the manganese content (*r* = 0.43) (Fig. 8B). Finally, 29 microbial variables were significantly correlated to mineral content in the roots, ranging from − 0.48 to 0.4. Interestingly, the calcium content was negatively correlated with the richness of bacteria (*r* = − 0.42), fungi (*r* = − 0.48), and AMF (*r* = − 0.42) in the rhizosphere. The manganese was also correlated with the AMF diversity (*r* = 0.4) and richness (*r* = 0.37) in the roots (Fig. 8C).

## Discussion

### The root compartments have distinct microbial communities which play different functions

Grapevine bacterial microbiome was largely composed of *Proteobacteria* and *Actinobacteria*. *Proteobacteria* are related to a wide range of functions involved in carbon, nitrogen, and sulfur cycling [47]. *Actinobacteria* are known for their production of secondary metabolites, degradation of organic substances and contribution to plant fitness by acting as PGPR or biocontrol agents [48]. Relatively similar proportions of bacterial phyla were found in other studies [24, 48, 49]. Interestingly, some studies found a greater abundance of *Acidobacteria* than *Actinobacteriota*, and observed several phyla such as *Armatimonadetes*, *Deinococcus*-*Thermus*, *Rokubacterua*, or *Synergistetes*, which were not detected in the GreffAdapt plot [23, 26]. Fungal microbiome was primarily dominated by *Ascomycota (Sordariomycetes, Dothideomycetes, Eurotiomycetes and Leotiomycetes)* and *Basidiomycota (Agaricomycetes, Tremellomycetes)*. These phyla have diverse ecological roles, such as ectomycorrhizae, plant pathogens and saprotrophic fungi, and are involved in nutrient acquisition, decomposition, and carbon sequestration [50, 51]. Several studies have found the same major fungal phyla and classes in a relatively similar proportion depending on the class [23, 24, 48]. Investigation of AMF community using 28S rRNA gene sequencing displayed a clear dominance of the *Glomus* genera in the rhizosphere and the root endosphere, which is consistent with other studies [52, 53]. Together, these results show that a core of bacteria and fungi is always recruited by grapevine root system compartments regardless of different environmental conditions. The highly dependent segregation of bacterial and fungal communities according to root system compartments, the enrichment of several taxa in both compartments, as well as the lower diversity and higher richness in the rhizosphere confirmed the plant’s ability to recruit only certain strains that will be able to reach the root endosphere [24]. Interestingly, the abundances of bacterial metabolic pathways necessary for plant development were significantly higher in the root endosphere, such as lipid metabolism, other amino acid metabolism and xenobiotics biodegradation and metabolism. Moreover, the fungal proportion of symbiotroph (such as arbuscular mycorrhizal) increased in the root endosphere, while that of pathotroph decreased (e.g., plant pathogen). These observations are consistent with the “cry for help” hypothesis, suggesting that the plant interacts with its surrounding microbial communities to alleviate different stresses by natural microbiome selection of specialized strains [54]. These differences could be explained by environmental conditions, such as temperature, humidity, and incidence of light, within both root endosphere and rhizosphere compartments, which result in different selection pressures for microorganisms [8].

### Rootstock genotypes drive the microbial communities in the root system

The level of fungi and archaea measured in the rhizosphere by qPCR is dependent on the rootstock genotype. Archaea offer promising applications for agriculture due to their important role in nutrient cycling, their various plant growth-promoting attributes, and their enhancement of tolerance to various abiotic stresses [55]. Despite the existence of a core microbiome between rootstocks, the genotype influenced at least one of the two ⍺-diversity metrics and the Bray-Curtis index measured for bacteria and fungi in both root system compartments. Several studies found the same effects, depending on the diversity indicator examined [22, 24, 26]. The enrichment of several taxa of bacteria and fungi in each rootstock genotype in the rhizosphere and the root endosphere suggested that rootstock associates preferentially with certain microbial composition, leading to various inferred metabolic functions. We can hypothesize that rhizodeposit composition varies between genotypes and that they recruit different microorganisms [15]. In grapevines, root exudates are still poorly characterized. Marastoni et al. (2020) unveiled common and specific responses in root exudate composition between Ramsey and 140R rootstocks subjected to iron deficiency [56]. Concerning AMF communities, all the ⍺-diversity metrics were influenced by the rootstock genotype in the root endosphere. In terms of β-diversity, it was difficult to draw any conclusions about the effect of the genotype. The Bray-Curtis index was significantly dependent on the rootstock genotype in both root system compartments and PCoA did not detect any clustering related to rootstock. Finally, few AMF taxa were enriched in several genotypes. These results confirms those obtained by Moukarzel et al. (2021) who showed, using denaturing gradient gel electrophoresis (DGGE) and trap cultures, that rootstock genotypes drive AMF community in the root system [29]. Previous work suggested differential production of strigolactones between RGM and 1103P [57]. We have just recently characterized the chemical structure of two non-canonical strigolactones in the exudates of these two rootstocks grown on nitrogen depleted medium [58]. Interestingly, RGM and 1103P exuded a similar concentration of vitislactone, whereas a heliolactone-like compound was detected exclusively in the root exudates of RGM. We can hypothesize that genotypes regulate the AMF community through the quantity and the types of strigolactones exuded into the rhizosphere. Finally, even if the soil physicochemical parameters were similar between the two sampled areas, an effect of the block was reported on several diversity indicators measured on bacterial, fungal and AMF communities. However, its contribution was generally lower than that of the genotype. Indeed, soil properties such as texture, structure, mineral content, and acidity affect microbial communities [59, 60], and are known to be one of the most important factors influencing microbial communities [24, 28].

### Scion genotype is involved in the selection of root system bacterial and fungal communities but with a smaller contribution than that of the rootstock

Studies have shown that the scion genotype influences the development of the rootstock root system [61] and that several signaling molecules are exchanged between the two components of the chimeric plant [1]. We therefore decided to study the effect of the scion genotype on the root system microbial communities with five scions grafted onto RGM. We retained this rootstock because we have already studied it in previous works [28, 58]. An effect of the scion genotype was reported on the level of bacteria, fungi and archaea measured in the rhizosphere. Among the ⍺-diversity metrics measured for the three groups of microorganisms, the scion genotype influenced the Simpson index measured for the bacteria in the rhizosphere only. Moreover, several microbial taxa were enriched in the root system of RGM grafted with different scion genotypes, and all the Bray-Curtis indexes were influenced by the scion genotype, with the exception of those measured for AMF in the root endosphere. Marasco et al. (2022) similarly found a contribution of the scion genotype towards the β-diversity of bacteria and fungi in the rhizosphere and the root tissue [24]. In addition, Vink et al. (2021) detected a scion effect on the Simpson and Faith’s PD indexes measured for bacterial community in the rhizosphere [26]. Dries et al. (2023) has just recently uncovered significant differences in α- and β-diversities between the scion genotypes Riesling and Mueller-Thurgau when cultivated ungrafted but were no longer observed when the scion were compared grafted [62]. Together, these results suggest that the scion genotype is involved in the selection of root system microbiomes. The scion is responsible for the production of sugars through photosynthesis and for their allocation to the rootstock [1]. If fewer sugars reach the rootstock, this could interfere with the sugar composition and quantity released into the rhizosphere, which undoubtedly mediate the functional and taxonomic diversity of microbial communities. In this case, the contribution of the scion genotype was less important than that of the rootstock genotype on the shaping of microbial communities. It would be interesting here to study the five scions grafted on another rootstock to see if we can repeat our results. Finally, our experimental design does not allow us to compare the contribution of the rootstock and the scion genotypes on the root system microbial communities together. However, the number of diversity metrics or bacterial pathways significantly influenced by the genotype with the associate PVE, as well as the number of microbial taxa enriched between the scion genotypes, suggest a lower involvement of the scion genotype than rootstock in the recruitment of microbial communities in the root system.

### Methodological considerations and future prospects

Instead of targeting one microbial community, multiplex sequencing of 3 amplicons provides a good alternative for a more global view of a microbial environment [28]. The use of the primers pair 341 F/785R is well suited to study soil bacteria. However, it amplifies a large proportion of host mitochondrial and chloroplastic 16S rRNA genes when studying plant tissues. Results on the root endosphere bacteria must be interpreted carefully because of the low sequencing depth obtained after removing host sequences and data rarefaction. Using pPNA and mPNA clamps to reduce host chloroplasts and mitochondria amplification is recommended for this type of study [63]. In our case, the use of the nested PCR could affect AMF community analysis, resulting in a decrease of taxa and diversity [64]. The comparisons between this method and classic PCR, as well as finding optimal primer pairs, must be carried out to arrive at a consensus on the method to analyze AMF community [65]. Quantifying AMF colonization of plant roots by microscopy or qPCR would have been interesting to compare the mycorrhization capacity of the root system between genotypes [66].

Although metabarcoding data cannot be used to study the role of microbiomes, it is possible to obtain an overview of microbial functions using taxonomy-based function prediction methods. These methods are based on the association of taxonomic clades with known functional guilds, or with metabolic functions or pathways obtained from public databases of annotated genomes [67]. Even though we were satisfied with PICRUSt2 results, the FUNGuild tool needs to be improved because more than half of the sequences obtained were not affiliated.

To our knowledge, this work is the first to explore correlations between microbial variables and plant phenotypic traits or mineral nutrition in grapevines, confirming the role of the root system microbiomes in grapevine development. It would be interesting to supplement these functional analyses in the rhizosphere by measuring the activities of microbial enzymes involved in soil biogeochemical cycling or by using community-level physiological profiling approach such as Biolog EcoPlate™ [68, 69]. Investigating another scion grafted onto all the rootstock genotypes studied would enable us to explore the influence of the rootstock-scion interaction on the belowground microbiomes, especially as it has been suggested that the rootstock–scion combination results were more significant than the two components taken alone [24]. Finally, coupling microbiome studies with biochemical analysis of the rhizosphere and the root endosphere could provide new insights into the understanding of plant-microorganism interactions.

## Conclusion

Our results confirm that rootstock genotypes drive the composition and the structure of bacteria and fungi microbiomes in the rhizosphere and the root endosphere. We showed that the scion genotypes have an influence on these communities, but to a lesser extent than the rootstock. We have also demonstrated that the different microbial communities observed between cultivars (i.e., rootstock or scion genotypes) may have different roles in their ecosystems. Moreover, this is the first study based on a metabarcoding approach which displays the rootstock genotype’s influence on the AMF community of grapevine root system, primarily in the root endosphere, while the involvement of the scion genotype remains disputable, depending on the diversity metrics observed (⍺ or β). Finally, we have highlighted correlations between the composition of the root system microbiomes and the phenotype and the mineral status of the plant, confirming the important role of these microorganisms in grapevine development.

### Electronic supplementary material

Below is the link to the electronic supplementary material.

**Additional file 1: Fig. S1**. Comparison of the relative abundances of bacterial phyla and classes, fungal phyla and classes, and AMF genera, between the root endosphere and the rhizosphere. **Fig. S2**. Venn diagrams showing common and specific OTUs of bacteria, fungi and AMF, between root compartments, rootstock and scion genotypes. **Fig. S3**. Histograms of the linear discriminant analysis (LDA) scores (> 4) reveal the most differentially abundant taxa of bacteria, fungi and AMF among the rhizosphere and the root endosphere. **Fig. S4**. Comparison of the levels of rhizosphere bacteria, fungi and archaea measured by qPCR between the rootstock or the scion genotypes. **Fig. S5**. Comparison of bacterial communities in the rhizosphere and the root endosphere between the 5 scion genotypes grafted onto RGM. **Fig. S6**. Histogram of the linear discriminant analysis (LDA) scores reveals the most differentially abundant taxa of bacteria, fungi and AMF in the rhizosphere and the root endosphere between scion genotypes grafted onto RGM. **Fig. S7**. Comparison of fungal communities in the rhizosphere and the root endosphere between the 5 scion genotypes grafted onto RGM. **Fig. S8**. Comparison of AMF communities in the rhizosphere and the root endosphere between the 5 scion genotypes grafted onto RGM. **Fig. S9**. PCA analysis of the predicted functions of bacteria (119 variables) between rootstock genotypes in the rhizosphere and the root endosphere. **Fig. S10**. Comparison between scion genotypes grafted onto RGM of the predicted trophic mode and guild in the rhizosphere and the root endosphere

**Additional file 2: Table S1**. Rootstocks and scion cultivars used in this study. **Table S2**. Soil analysis carried out on the block 2 and 3 of the GreffAdapt plot. **Table S3** List of primers and PCR conditions used for the metabarcoding approaches. **Table S4**. Quantification of the rhizosphere bacteria and fungi with the cultivable approach, and the rhizosphere bacteria, fungi and archaea with qPCR. **Table S5**. Pairwise Adonis comparisons of the Bray-Curtis index measured for bacterial community between the genotypes of rootstock or scion in the rhizosphere and the root endosphere. **Table S6**. Pairwise Adonis comparisons of the Bray-Curtis index measured for fungal community between the genotypes of rootstock or scion in the rhizosphere and the root endosphere. **Table S7**. Pairwise Adonis comparisons of the Bray-Curtis index measured for AMF community between the genotypes of rootstock or scion in the rhizosphere and the root endosphere. **Table S8**. Comparison of the abundances of the bacterial metabolic pathways predicted with PICRUST2 between the rhizosphere and the root endosphere. **Table S9**. Comparison of the abundances of the bacterial metabolic pathways predicted with PICRUST2 between rootstock or scion genotypes in the rhizosphere or the root endosphere

## Data Availability

The data for this study have been deposited in the European Nucleotide Archive (ENA) at EMBL-EBI under accession number PRJEB65486 (https://www.ebi.ac.uk/ena/browser/view/PRJEB65486).
