# Supplementary material for: Both the scion and rootstock of grafted grapevines influence the rhizosphere and root endophyte microbiomes, but rootstocks have a greater impact
Source: Environ Microbiome. 2024 Apr 23;19:24. doi: 10.1186/s40793-024-00566-5 (PMC11040986; doi:10.1186/s40793-024-00566-5)
Supplement: Supplementary file 1 — Additional file 1: Fig. S1. Comparison of the relative abundances of bacterial phyla and classes, fungal phyla and classes, and AMF genera, between the root endosphere and the rhizosphere. Fig. S2. Venn diagrams showing common and specific OTUs of bacteria, fungi and AMF, between root compartments, rootstock and scion genotypes. Fig. S3. Histograms of the linear discriminant analysis (LDA) scores (> 4) reveal the most differentially abundant taxa of bacteria, fungi and AMF among the rhizosphere and the root endosphere. Fig. S4. Comparison of the levels of rhizosphere bacteria, fungi and archaea measured by qPCR between the rootstock or the scion genotypes. Fig. S5. Comparison of bacterial communities in the rhizosphere and the root endosphere between the 5 scion genotypes grafted onto RGM. Fig. S6. Histogram of the linear discriminant analysis (LDA) scores reveals the most differentially abundant taxa of bacteria, fungi and AMF in the rhizosphere and the root endosphere between scion genotypes grafted onto RGM. Fig. S7. Comparison of fungal communities in the rhizosphere and the root endosphere between the 5 scion genotypes grafted onto RGM. Fig. S8. Comparison of AMF communities in the rhizosphere and the root endosphere between the 5 scion genotypes grafted onto RGM. Fig. S9. PCA analysis of the predicted functions of bacteria (119 variables) between rootstock genotypes in the rhizosphere and the root endosphere. Fig. S10. Comparison between scion genotypes grafted onto RGM of the predicted trophic mode and guild in the rhizosphere and the root endosphere [file 40793_2024_566_MOESM1_ESM.docx]

**Additional file 1**


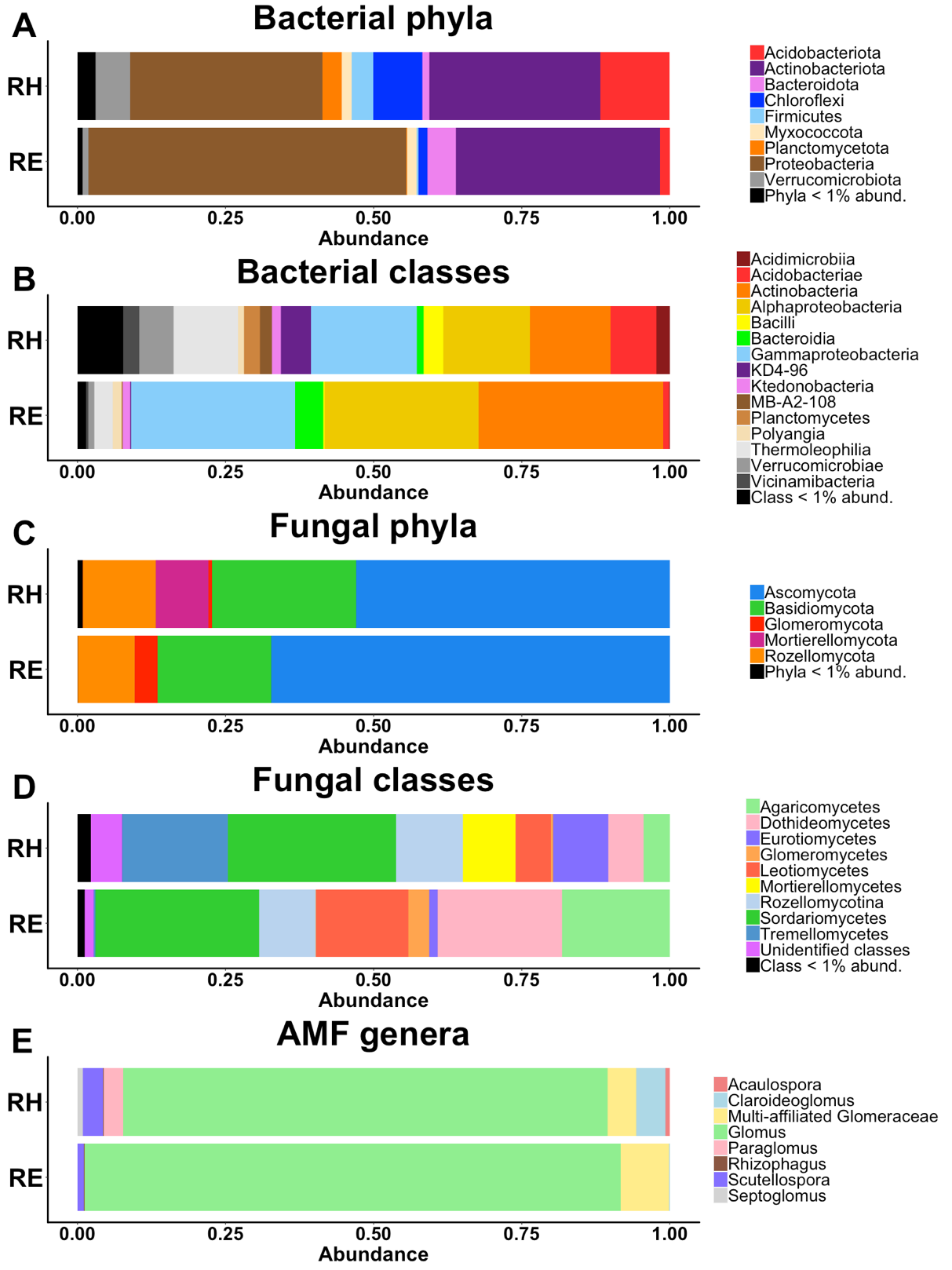


**Fig. S1** Comparison of the relative abundances of (**A**, **B**) bacterial phyla and classes, (**C**, **D**) fungal phyla and classes, and (**E**) AMF genera, between the root endosphere and the rhizosphere.

**Fig. S2** Venn diagrams showing common and specific OTUs of bacteria, fungi and AMF, between (**A**) root compartments, (**B**) rootstock and (**C**) scion genotypes.


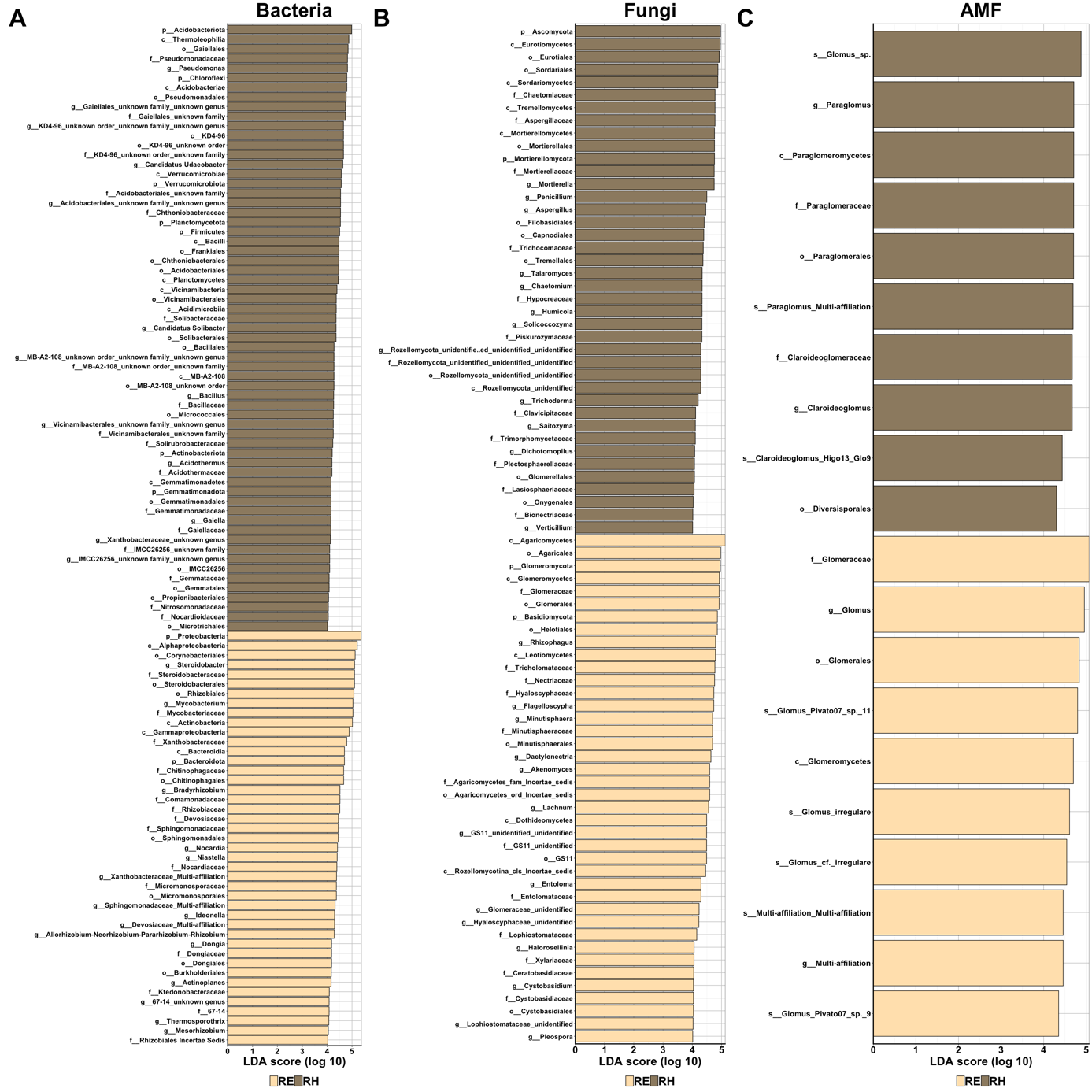


**Fig. S3** Histograms of the linear discriminant analysis (LDA) scores (> 4) reveal the most differentially abundant taxa of (**A**) bacteria, (**B**) fungi and (**C**) AMF among the rhizosphere (dark brown) and the root endosphere (pale brown).

**
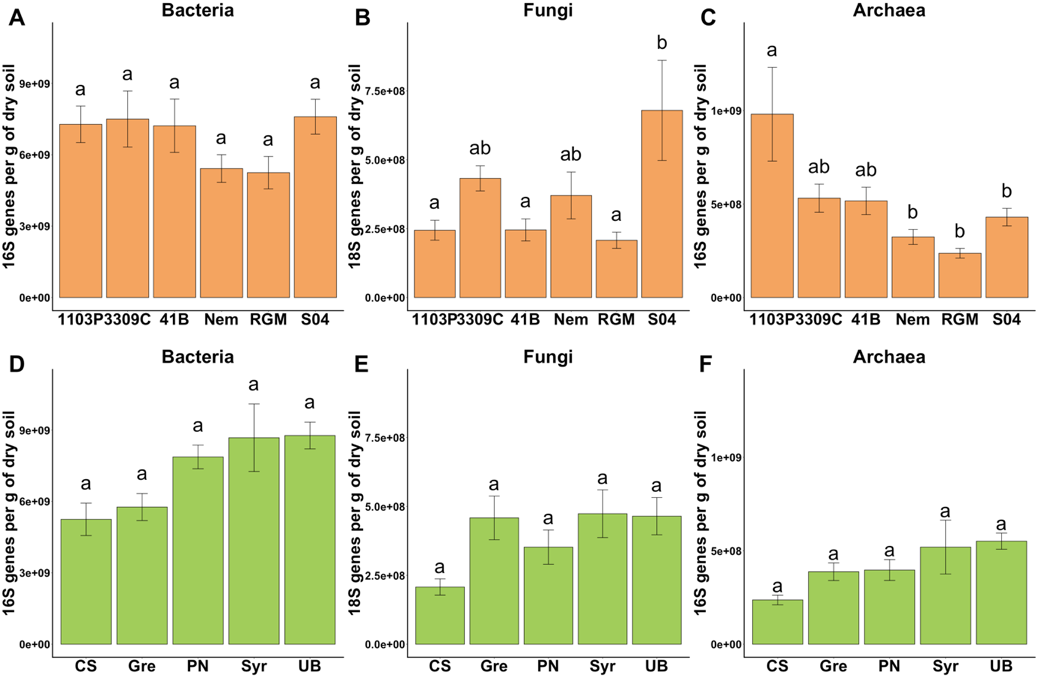
**

**Fig. S4** Comparison of the levels of rhizosphere bacteria, fungi and archaea measured by qPCR between the rootstock (**A**, **B** and **C**, respectively, orange) or the scion genotypes (**D**, **E** and **F**, respectively, green).

Data are represented as mean ± SE, for the comparisons between rootstocks (orange, n = 6) or scion genotypes (green, n = 5). *P*-values were calculated using pairwise-Student tests with Bonferroni correction and letters indicate significant differences when the adjusted *P*-value < 0.05.

**
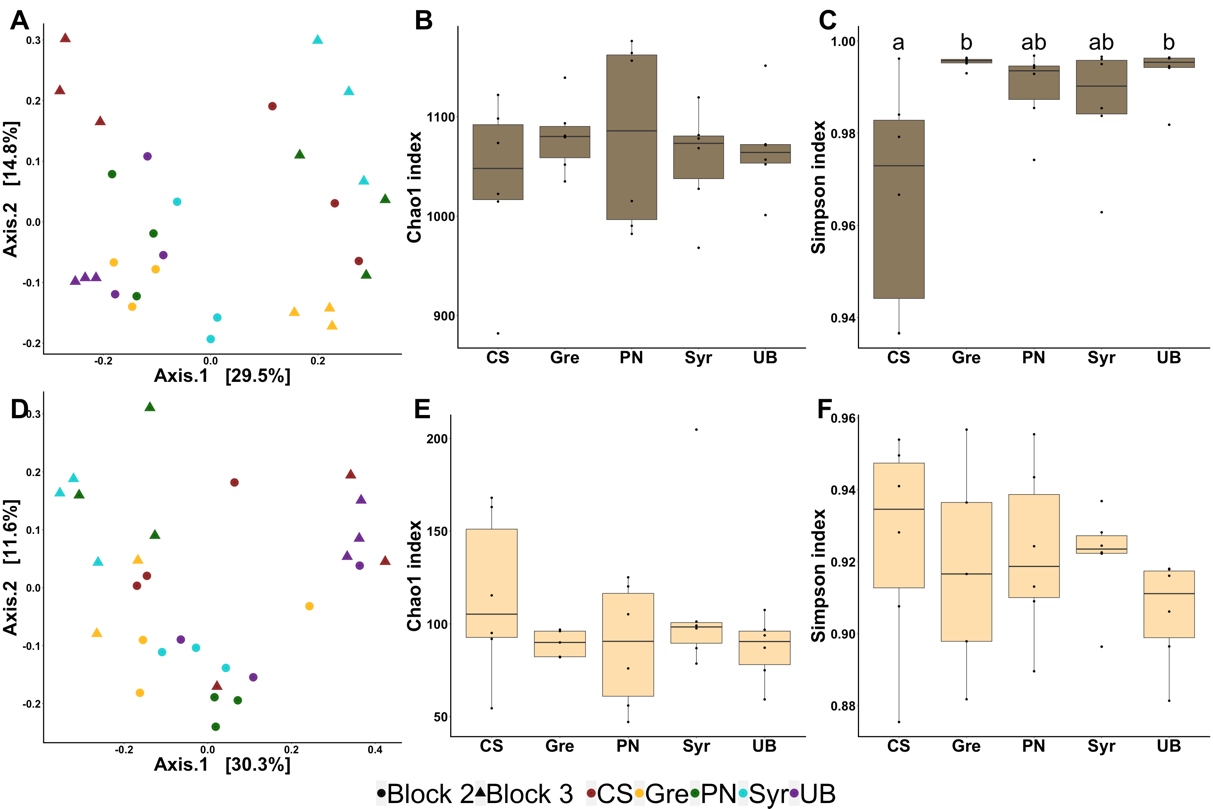
**

**Fig. S5** Comparison of bacterial communities in the rhizosphere (dark brown, top panel) and the root endosphere (light brown, bottom panel) between the 5 scion genotypes grafted onto RGM (n=6).

Comparison of bacterial communities by principal coordinate analysis (PCoA) based on Bray–Curtis dissimilarity matrices in the rhizosphere (**A)** and the root endosphere (**D)**. Comparison of bacterial richness (Chao1) and diversity (Simpson) in the rhizosphere (**B**, **C**) and the root endosphere (**E**, **F**). *p*-values were calculated using Pairwise-Student tests with Bonferroni correction and were considered as significant when the adjusted *P*-value < 0.05.

**
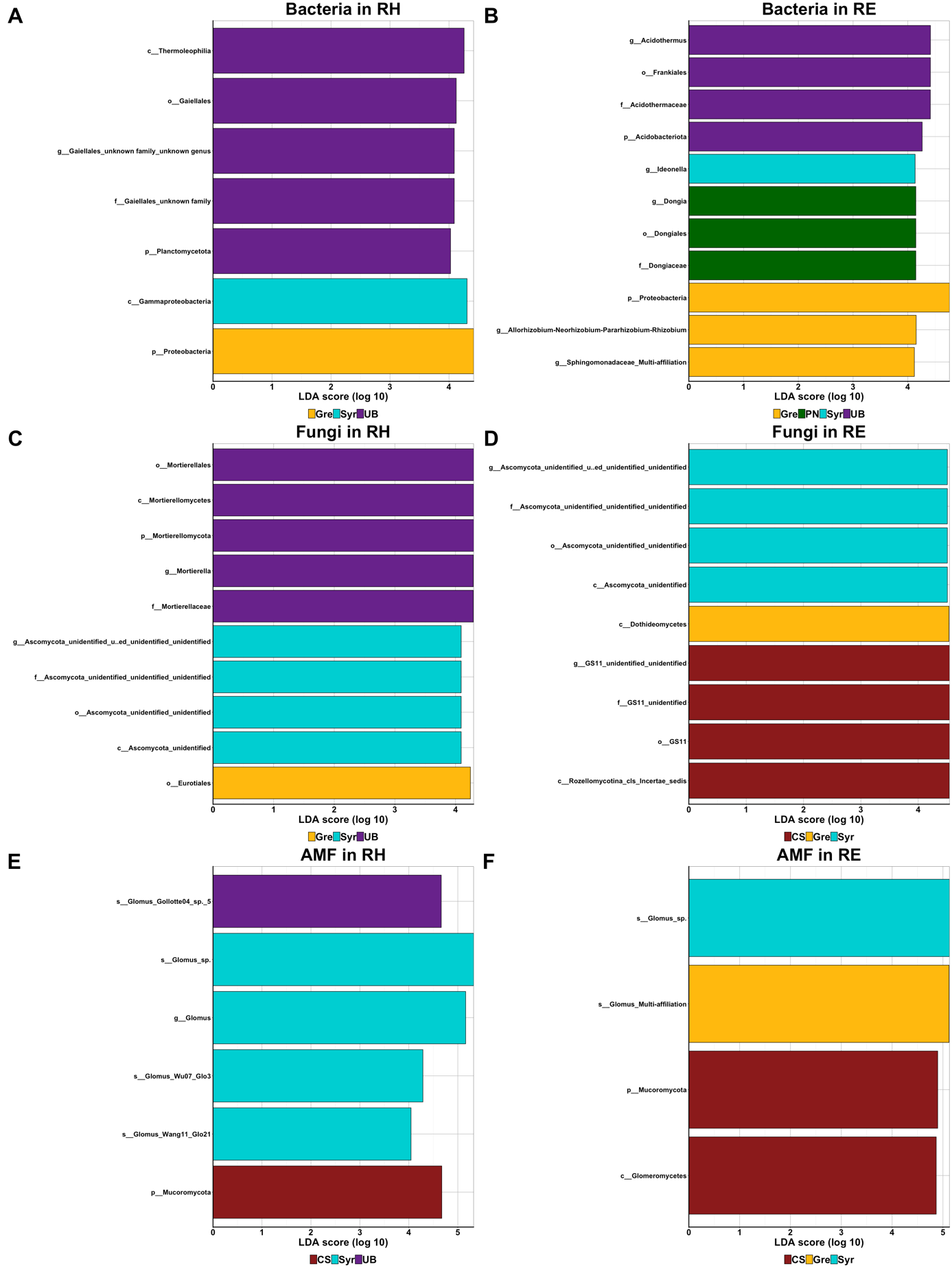
**

**Fig. S6** Histogram of the linear discriminant analysis (LDA) scores reveals the most differentially abundant taxa of bacteria (**A**, **B**), fungi (**C**, **D**) and AMF (**D**, **E**) in the rhizosphere (RH) and the root endosphere (RE) between scion genotypes grafted onto RGM.

**
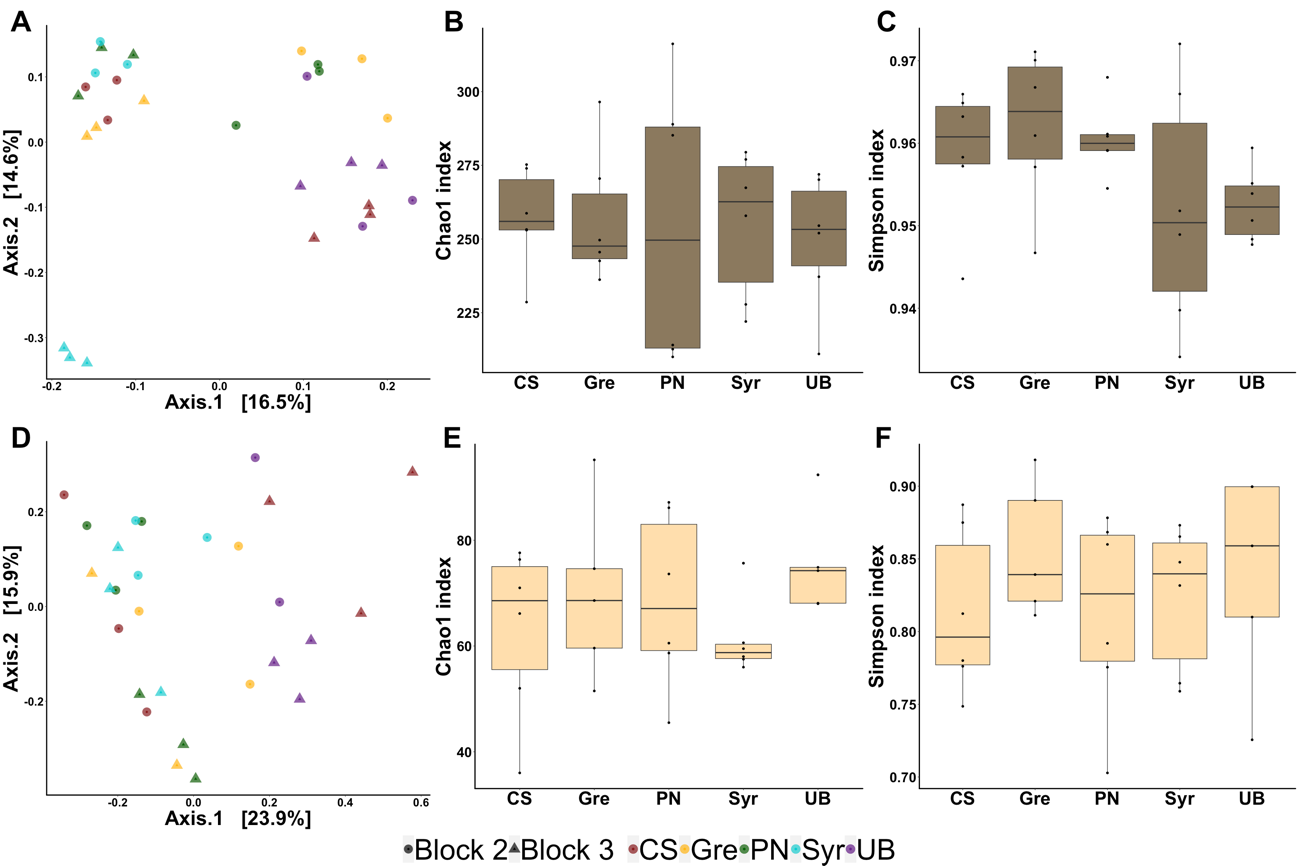
**

**Fig. S7** Comparison of fungal communities in the rhizosphere (dark brown, top panel) and the root endosphere (light brown, bottom panel) between the 5 scion genotypes grafted onto RGM (n=6).

Comparison of fungal communities by principal coordinate analysis (PCoA) based on Bray–Curtis dissimilarity matrices in the rhizosphere (**A)** and the root endosphere (**D)**. Comparison of fungal richness (Chao1) and diversity (Simpson) in the rhizosphere (**B**, **C**) and the root endosphere (**E**, **F**). *P*-values were calculated using Pairwise-Student tests with Bonferroni correction and were considered as significant when the adjusted *P*-value < 0.05.


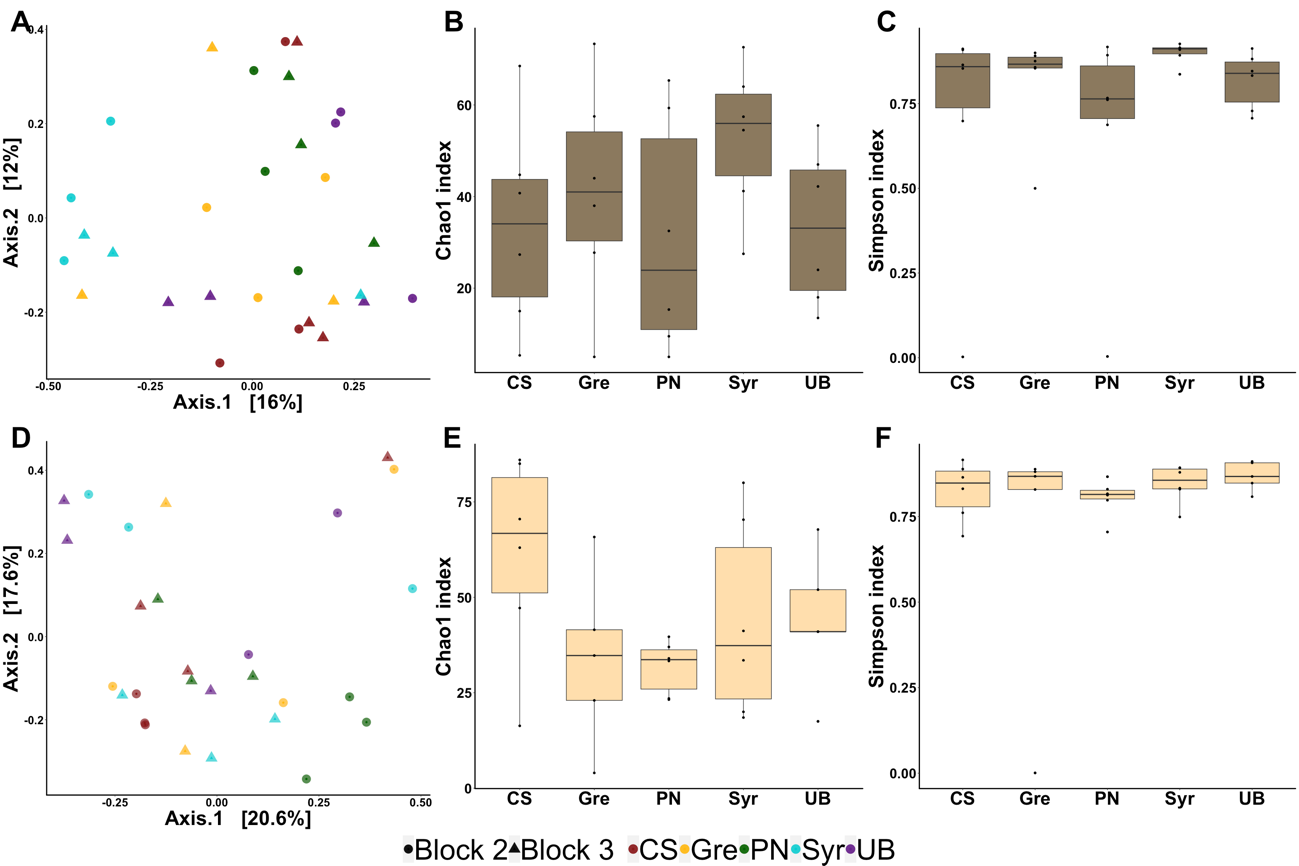


**Fig. S8** Comparison of AMF communities in the rhizosphere (dark brown, top panel) and the root endosphere (light brown, bottom panel) between the 5 scion genotypes grafted onto RGM (n=6).

Comparison of AMF communities by principal coordinate analysis (PCoA) based on Bray–Curtis dissimilarity matrices in the rhizosphere (**A)** and the root endosphere (**D)**. Comparison of AMF richness (Chao1) and diversity (Simpson) in the rhizosphere (**B**, **C**) and the root endosphere (**E**, **F**). *P*-values were calculated using Pairwise-Student tests with Bonferroni correction and were considered as significant when the adjusted *P*-value < 0.05.

**
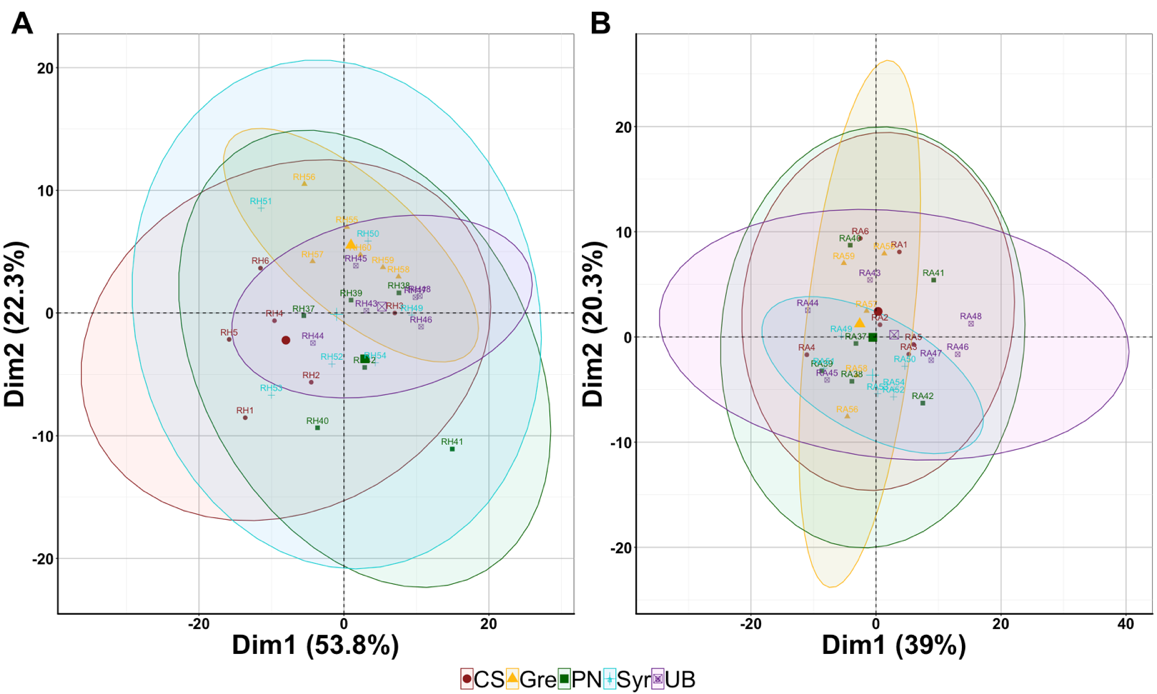
**

**Fig. S9** PCA analysis of the predicted functions of bacteria (119 variables) between rootstock genotypes in the rhizosphere (A) and the root endosphere (B).


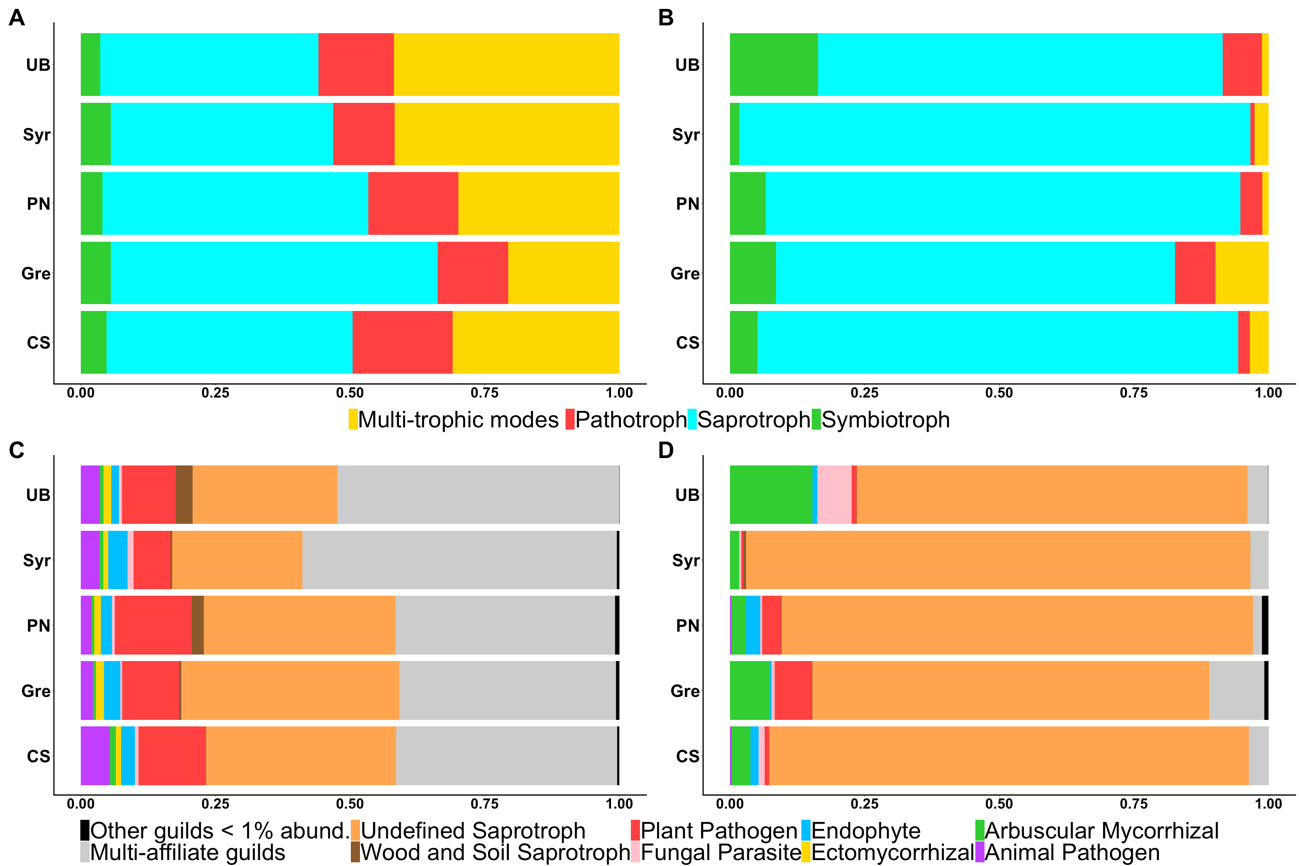


**Fig. S10** Comparison between scion genotypes grafted onto RGM of the predicted trophic mode and guild in the rhizosphere (**A**, **C**) and the root endosphere (**B**, **D**).
