# Supplementary material for: Both the scion and rootstock of grafted grapevines influence the rhizosphere and root endophyte microbiomes, but rootstocks have a greater impact
Source: Environ Microbiome. 2024 Apr 23;19:24. doi: 10.1186/s40793-024-00566-5 (PMC11040986; doi:10.1186/s40793-024-00566-5)
Supplement: Supplementary file 2 — Additional file 2: Table S1. Rootstocks and scion cultivars used in this study. Table S2. Soil analysis carried out on the block 2 and 3 of the GreffAdapt plot. Table S3 List of primers and PCR conditions used for the metabarcoding approaches. Table S4. Quantification of the rhizosphere bacteria and fungi with the cultivable approach, and the rhizosphere bacteria, fungi and archaea with qPCR. Table S5. Pairwise Adonis comparisons of the Bray-Curtis index measured for bacterial community between the genotypes of rootstock or scion in the rhizosphere and the root endosphere. Table S6. Pairwise Adonis comparisons of the Bray-Curtis index measured for fungal community between the genotypes of rootstock or scion in the rhizosphere and the root endosphere. Table S7. Pairwise Adonis comparisons of the Bray-Curtis index measured for AMF community between the genotypes of rootstock or scion in the rhizosphere and the root endosphere. Table S8. Comparison of the abundances of the bacterial metabolic pathways predicted with PICRUST2 between the rhizosphere and the root endosphere. Table S9. Comparison of the abundances of the bacterial metabolic pathways predicted with PICRUST2 between rootstock or scion genotypes in the rhizosphere or the root endosphere [file 40793_2024_566_MOESM2_ESM.docx]

**Additional file 2**

**Table S1** Rootstocks and scion cultivars used in this study.

|  | **Name** | **Genetic backgrounds** | **Characteristics** | |
| --- | --- | --- | --- | --- |
|  |  |  | Conferred vigour | Water deficit tolerance |
| **Rootstocks grafted with Cabernet Sauvignon** | Selection Oppenheim 4 (SO4) | *V. berlandieri × V. riparia* | High | Medium to good |
|  | 3309 Couderc (3309C) | *V. riparia × V. rupestris* | Low to medium | Weak in top soil |
|  | 41 B MGt (41B) | *V. berlandieri × V. vinifera* | Medium to high | Medium to high |
|  | 1103 Paulsen (1103P) | *V. berlandieri × V. rupestris* | High | Good |
|  | Riparia Gloire de Montpellier (RGM) | *V. riparia* | Very low to low | Weak |
|  | Nemadex AB | *V. berlandieri × V. Muscadinia × V. vinifera × V. rupestris* | Low | Weak |
| **Scion cultivars grafted on RGM** |  |  | Berry color |  |
|  | Cabernet-Sauvignon clone 169 | *V. vinifera* | Red |  |
|  | Syrah clone 524 | *V. vinifera* | Red |  |
|  | Grenache clone 362 | *V. vinifera* | Red |  |
|  | Pinot noir clone 113 | *V. vinifera* | Red |  |
|  | Ugni blanc clone 481 | *V. vinifera* | White |  |

**Table S2** Soil analysis carried out on the block 2 and 3 of the GreffAdapt plot.

|  | **Block 2** | **Block 3** |
| --- | --- | --- |
| **Depth (cm)** | 0-35 | 0-30 |
| **Coarse elements (%)** | 31 | 33,6 |
| **Fine soil (%)** | 69 | 66,4 |
| **Coarse sands (‰)** | 761 | 738 |
| **Fine sands (‰)** | 82 | 120 |
| **Coarse silts (‰)** | 40 | 36 |
| **Fine silts (‰)** | 65 | 62 |
| **Clay (‰)** | 52 | 45 |
| **Texture given** | Sand | Sand |
| **Organic matter (%)** | 2,2 | 1,2 |
| **Total nitrogen (%)** | 0,109 | 0,049 |
| **C / N** | 11,5 | 14,7 |
| **Water pH** | 7,2 | 6,7 |
| **Absorbent complex** | | |
| **Potassium (g / 100g)** | 0,28 | 0,71 |
| **Magnesium (g / 100g)** | 0,67 | 0,61 |
| **Calcium (g / 100g)** | 1,76 | 7,2 |
| **C.E.C. (cmol+ / kg)** | 5,3 | 3 |
| **Saturation (%)** | 127,1 | 102,4 |

**Table S3** List of primers and PCR conditions used for the metabarcoding approaches.

| **Target genes** | **Primers** | **Sequences (5'-3')** | **Initial denaturation** | **Number of cycles** | **Denaturation** | **Annealing** | **Extension** | **Final extension** | **Sources** |  |
| --- | --- | --- | --- | --- | --- | --- | --- | --- | --- | --- |
|  |  |  |  |  |  |  |  |  |  |  |
| **Bacterial 16S rRNA gene V3-V4 regions** | 341F | CCTACGGGNGGCWGCAG | 95°C/5min | 25 | 95°C/30s | 55°C/30s | 72°C/30s | 72°C/5min | Klindworth et al., 2013 |  |
|  | 785R | GACTACHVGGGTATCTAATCC |  |  |  |  |  |  |  |  |
| **Fungal ITS1 region (highly variable)** | ITS1F | CTTGGTCATTTAGAGGAAGTAA | 95°C/5min | 30 | 95°C/30s | 55°C/30s | 72°C/45s | 72°C/7min | Gardes and Bruns, 1993 White et al., 1990 |  |
|  | ITS2 | GCTGCGTTCTTCATCGATGC |  |  |  |  |  |  |  |  |
| **Eurayote 28S rRNA gene** | LR1 | GCATATCAATAAGCGGAGGA | 95°C/5min | 30 | 95°C/30s | 58°C/30s | 72°C/30s | 72°C/5min | van Tuinen et al., 1998 |  |
|  | NDL22 | TGGTCCGTGTTTCAAGACG |  |  |  |  |  |  |  |  |
| **AMF 28S rRNA gene** | FLR3 | TTGAAAGGGAAACGATTGAAGT | 98°C/30s | 30 | 95°C/30s | 55°C/30s | 72°C/45s | 72°C/7min | Gollotte et al. 2004 |  |
|  | FLR4 | TACGTCAACATCCTTAACGAA |  |  |  |  |  |  |  |  |
| **Specific overhang Illumina adapters** | Forward | TCGTCGGCAGCGTCAGATGTGTATAAGAGACAG |  |  |  |  |  |  | PGTB |  |
|  | Reverse | GTCTCGTGGGCTCGGAGATGTGTATAAGAGACAG |  |  |  |  |  |  |  |  |

**Table S4** Quantification of the rhizosphere bacteria and fungi with the cultivable approach, and the rhizosphere bacteria, fungi and archaea with qPCR.

|  |  | **Cultivable** | | | | | | **qPCR** | | | | | | | | |
| --- | --- | --- | --- | --- | --- | --- | --- | --- | --- | --- | --- | --- | --- | --- | --- | --- |
|  |  | **Bacteria** | | | **Fungi** | | | **Bacteria** | | | **Fungi** | | | **Archaea** | | |
|  |  | ***p*-value** | | **PVE** | ***p*-value** | | **PVE** | ***p*-value** | | **PVE** | ***p*-value** | | **PVE** | ***p*-value** | | **PVE** |
| **Rootstock** | **Genotype (G)** | 0.296 | ns | / | 0.118 | ns | / | 0.218 | ns | / | 0.004 | ****** | **41%** | **8.96e-04** | ******* | **46%** |
|  | **Bloc (B)** | 0.145 | ns | / | 0.179 | ns | / | 0.432 | ns | / | 0.065 | ns | / | 0.054 | ns | / |
|  | **G x B** | 0.341 | ns | / | **0.0172** | ***** | **30%** | 0.342 | ns | / | 0.052 | ns | / | **3.28e-05** | ******* | **32%** |
| **Scion** | **G** | 0.109 | ns | / | 0.135 | ns | / | **0.007** | ****** | **39%** | **0.0357** | ***** | **31%** | **0.016** | ***** | **30%** |
|  | **B** | 0.465 | ns | / | 0.645 | ns | / | 0.067 | ns | / | 0.0821 | ns | / | **0.003** | ****** | **22%** |
|  | **G x B** | 0.566 | ns | / | 0.287 | ns | / | 0.465 | ns | / | 0.1479 | ns | / | **0.002** | ****** | **27%** |

**Table S5** Pairwise Adonis comparisons of the Bray-Curtis index measured for bacterial community between the genotypes of rootstock or scion in the rhizosphere and the root endosphere.

|  |  | **Bacteria** | | | | | | | | | | | |
| --- | --- | --- | --- | --- | --- | --- | --- | --- | --- | --- | --- | --- | --- |
|  |  | **Rhizosphere** | | | | | | **Root endosphere** | | | | | |
|  |  | **Df** | **SumsOfSqs** | **F.Model** | **R2** | ***p*-value** | ***p*-adjusted** | **Df** | **SumsOfSqs** | **F.Model** | **R2** | ***p*-value** | ***p*-adjusted** |
| **Rootstock** | **1103P vs Nem** | 1 | 1.373 | 17.56 | 0.637 | 0.002 | **0.03** | 1 | 1.379 | 9.45 | 0.486 | 0.001 | **0.015** |
|  | **1103P vs SO4** | 1 | 1.132 | 12.44 | 0.554 | 0.002 | **0.03** | 1 | 1.322 | 9.96 | 0.499 | 0.005 | 0.075 |
|  | **1103P vs RGM** | 1 | 0.969 | 8.46 | 0.458 | 0.003 | **0.045** | 1 | 0.921 | 5.35 | 0.346 | 0.002 | **0.03** |
|  | **1103P vs 41B** | 1 | 0.468 | 5.49 | 0.355 | 0.002 | **0.03** | 1 | 0.600 | 4.47 | 0.309 | 0.003 | **0.045** |
|  | **1103P vs 3309C** | 1 | 1.329 | 16.71 | 0.626 | 0.002 | **0.03** | 1 | 1.295 | 8.65 | 0.464 | 0.003 | **0.045** |
|  | **Nem vs SO4** | 1 | 0.202 | 2.10 | 0.174 | 0.042 | 0.63 | 1 | 0.273 | 1.97 | 0.165 | 0.009 | 0.135 |
|  | **Nem vs RGM** | 1 | 0.361 | 3.02 | 0.232 | 0.013 | 0.195 | 1 | 0.350 | 1.96 | 0.164 | 0.028 | 0.42 |
|  | **Nem vs 41B** | 1 | 0.860 | 9.53 | 0.488 | 0.001 | **0.015** | 1 | 0.749 | 5.34 | 0.348 | 0.005 | 0.075 |
|  | **Nem vs 3309C** | 1 | 0.122 | 1.45 | 0.126 | 0.099 | 1 | 1 | 0.414 | 2.66 | 0.210 | 0.008 | 0.12 |
|  | **SO4 vs RGM** | 1 | 0.230 | 1.739 | 0.148 | 0.113 | 1 | 1 | 0.296 | 1.79 | 0.152 | 0.043 | 0.645 |
|  | **SO4 vs 41B** | 1 | 0.545 | 5.30 | 0.346 | 0.003 | **0.045** | 1 | 0.691 | 5.44 | 0.352 | 0.007 | 0.105 |
|  | **SO4 vs 3309C** | 1 | 0.137 | 1.41 | 0.123 | 0.149 | 1 | 1 | 0.350 | 2.45 | 0.197 | 0.007 | 0.105 |
|  | **RGM vs 41B** | 1 | 0.486 | 3.84 | 0.277 | 0.015 | 0.225 | 1 | 0.304 | 1.83 | 0.155 | 0.025 | 0.375 |
|  | **RGM vs 3309C** | 1 | 0.275 | 2.28 | 0.186 | 0.02 | 0.3 | 1 | 0.209 | 1.15 | 0.103 | 0.319 | 1 |
|  | **41B vs 3309C** | 1 | 0.749 | 8.19 | 0.450 | 0.003 | **0.045** | 1 | 0.569 | 3.95 | 0.283 | 0.003 | **0.045** |
| **Scion** | **CS vs PN** | 1 | 0.165 | 1.12 | 0.101 | 0.241 | 1,000 | 1 | 0.181 | 0.95 | 0.087 | 0.489 | 1 |
|  | **CS vs UB** | 1 | 0.323 | 2.77 | 0.217 | 0.013 | 0.13 | 1 | 0.233 | 1.33 | 0.117 | 0.178 | 1 |
|  | **CS vs Syr** | 1 | 0.198 | 1.37 | 0.120 | 0.238 | 1 | 1 | 0.307 | 1.79 | 0.152 | 0.058 | 0.58 |
|  | **CS vs Gre** | 1 | 0.295 | 2.21 | 0.181 | 0.074 | 0.74 | 1 | 0.254 | 1.46 | 0.140 | 0.143 | 1 |
|  | **PN vs UB** | 1 | 0.286 | 2.65 | 0.210 | 0.01 | 0.1 | 1 | 0.433 | 2.71 | 0.213 | 0.009 | 0.09 |
|  | **PN vs Syr** | 1 | 0.146 | 1.07 | 0.097 | 0.344 | 1 | 1 | 0.135 | 0.87 | 0.080 | 0.551 | 1 |
|  | **PN vs Gre** | 1 | 0.172 | 1.38 | 0.121 | 0.126 | 1 | 1 | 0.130 | 0.83 | 0.084 | 0.597 | 1 |
|  | **UB vs Syr** | 1 | 0.440 | 4.17 | 0.294 | 0.004 | **0.04** | 1 | 0.633 | 4.46 | 0.308 | 0.003 | **0.03** |
|  | **UB vs Gre** | 1 | 0.364 | 3.88 | 0.279 | 0.003 | **0.03** | 1 | 0.471 | 3.33 | 0.270 | 0.022 | 0.22 |
|  | **Syr vs Gre** | 1 | 0.222 | 1.81 | 0.153 | 0.105 | 1 | 1 | 0.176 | 1.28 | 0.125 | 0.221 | 1 |

**Table S6** Pairwise Adonis comparisons of the Bray-Curtis index measured for fungal community between the genotypes of rootstock or scion in the rhizosphere and the root endosphere.

|  |  | **Fungi** | | | | | | | | | | | |
| --- | --- | --- | --- | --- | --- | --- | --- | --- | --- | --- | --- | --- | --- |
|  |  | **Rhizosphere** | | | | | | **Root endosphere** | | | | | |
|  |  | **Df** | **SumsOfSqs** | **F.Model** | **R2** | ***p*-value** | ***p*-adjusted** | **Df** | **SumsOfSqs** | **F.Model** | **R2** | ***p*-value** | ***p*-adjusted** |
| **Rootstock** | **1103P vs Nem** | 1 | 0.973 | 8.44 | 0.458 | 0.001 | **0.015** | 1 | 0.943 | 3.46 | 0.278 | 0.002 | **0.03** |
|  | **1103P vs SO4** | 1 | 0.964 | 7.32 | 0.423 | 0.003 | **0.045** | 1 | 0.938 | 3.89 | 0.302 | 0.004 | 0.06 |
|  | **1103P vs RGM** | 1 | 0.839 | 7.13 | 0.416 | 0.002 | **0.03** | 1 | 0.594 | 2.03 | 0.184 | 0.054 | 0.81 |
|  | **1103P vs 41B** | 1 | 0.574 | 4.81 | 0.325 | 0.004 | 0.06 | 1 | 0.848 | 3.80 | 0.297 | 0.001 | **0.015** |
|  | **1103P vs 3309C** | 1 | 0.987 | 10.00 | 0.500 | 0.003 | **0.045** | 1 | 1.022 | 4.07 | 0.311 | 0.003 | **0.045** |
|  | **Nem vs SO4** | 1 | 0.380 | 2.90 | 0.225 | 0.02 | 0.3 | 1 | 0.322 | 1.54 | 0.133 | 0.128 | 1 |
|  | **Nem vs RGM** | 1 | 0.190 | 1.62 | 0.140 | 0.095 | 1 | 1 | 0.400 | 1.56 | 0.135 | 0.098 | 1 |
|  | **Nem vs 41B** | 1 | 0.495 | 4.18 | 0.295 | 0.003 | **0.045** | 1 | 0.764 | 3.94 | 0.283 | 0.003 | **0.045** |
|  | **Nem vs 3309C** | 1 | 0.156 | 1.59 | 0.137 | 0.013 | 0.195 | 1 | 0.353 | 1.62 | 0.139 | 0.093 | 1 |
|  | **SO4 vs RGM** | 1 | 0.312 | 2.34 | 0.190 | 0.032 | 0.48 | 1 | 0.528 | 2.31 | 0.187 | 0.032 | 0.48 |
|  | **SO4 vs 41B** | 1 | 0.416 | 3.08 | 0.235 | 0.008 | 0.12 | 1 | 0.419 | 2.53 | 0.202 | 0.01 | 0.15 |
|  | **SO4 vs 3309C** | 1 | 0.303 | 2.64 | 0.209 | 0.051 | 0.765 | 1 | 0.453 | 2.38 | 0.192 | 0.036 | 0.54 |
|  | **RGM vs 41B** | 1 | 0.343 | 2.84 | 0.221 | 0.005 | 0.075 | 1 | 0.734 | 3.45 | 0.256 | 0.004 | 0.06 |
|  | **RGM vs 3309C** | 1 | 0.263 | 2.62 | 0.208 | 0.001 | **0.015** | 1 | 0.334 | 1.41 | 0.123 | 0.19 | 1 |
|  | **41B vs 3309C** | 1 | 0.471 | 4.62 | 0.316 | 0.001 | **0.015** | 1 | 0.891 | 5.09 | 0.337 | 0.002 | **0.03** |
| **Scion** | **CS vs PN** | 1 | 0.181 | 1.65 | 0.141 | 0.104 | 1 | 1 | 0.409 | 1.79 | 0.151 | 0.114 | 1 |
|  | **CS vs UB** | 1 | 0.243 | 2.37 | 0.192 | 0.011 | 0.11 | 1 | 0.317 | 1.42 | 0.136 | 0.198 | 1 |
|  | **CS vs Syr** | 1 | 0.284 | 2.18 | 0.179 | 0.02 | 0.2 | 1 | 0.334 | 1.39 | 0.122 | 0.208 | 1 |
|  | **CS vs Gre** | 1 | 0.213 | 1.77 | 0.150 | 0.063 | 0.63 | 1 | 0.295 | 1.25 | 0.121 | 0.266 | 1 |
|  | **PN vs UB** | 1 | 0.314 | 3.38 | 0.252 | 0.002 | **0.02** | 1 | 0.546 | 3.19 | 0.262 | 0.002 | **0.02** |
|  | **PN vs Syr** | 1 | 0.376 | 3.11 | 0.237 | 0.007 | 0.07 | 1 | 0.340 | 1.75 | 0.149 | 0.049 | 0.49 |
|  | **PN vs Gre** | 1 | 0.235 | 2.12 | 0.175 | 0.006 | 0.06 | 1 | 0.191 | 1.04 | 0.103 | 0.367 | 1 |
|  | **UB vs Syr** | 1 | 0.445 | 3.91 | 0.281 | 0.003 | **0.03** | 1 | 0.541 | 2.94 | 0.246 | 0.006 | 0.06 |
|  | **UB vs Gre** | 1 | 0.321 | 3.11 | 0.237 | 0.001 | **0.01** | 1 | 0.381 | 2.20 | 0.216 | 0.022 | 0.22 |
|  | **Syr vs Gre** | 1 | 0.371 | 2.83 | 0.221 | 0.005 | **0.05** | 1 | 0.272 | 1.38 | 0.133 | 0.159 | 1 |

**Table S7** Pairwise Adonis comparisons of the Bray-Curtis index measured for AMF community between the genotypes of rootstock or scion in the rhizosphere and the root endosphere.

|  |  | **AMF** | | | | | | | | | | | |
| --- | --- | --- | --- | --- | --- | --- | --- | --- | --- | --- | --- | --- | --- |
|  |  | **Rhizosphere** | | | | | | **Root endosphere** | | | | | |
|  |  | **Df** | **SumsOfSqs** | **F.Model** | **R2** | ***p*-value** | ***p*-adjusted** | **Df** | **SumsOfSqs** | **F.Model** | **R2** | ***p*-value** | ***p*-adjusted** |
| **Rootstock** | **1103P vs Nem** | 1 | 0.750 | 1.99 | 0.166 | 0.011 | 0.165 | 1 | 0.541 | 1.62 | 0.153 | 0.075 | 1 |
|  | **1103P vs SO4** | 1 | 0.636 | 1.56 | 0.135 | 0.071 | 1 | 1 | 0.299 | 0.83 | 0.085 | 0.615 | 1 |
|  | **1103P vs RGM** | 1 | 0.534 | 1.46 | 0.127 | 0.162 | 1 | 1 | 0.512 | 1.68 | 0.157 | 0.064 | 0.96 |
|  | **1103P vs 41B** | 1 | 0.532 | 1.45 | 0.126 | 0.14 | 1 | 1 | 0.478 | 1.13 | 0.112 | 0.257 | 1 |
|  | **1103P vs 3309C** | 1 | 1.047 | 3.35 | 0.251 | 0.002 | **0.03** | 1 | 0.587 | 1.80 | 0.167 | 0.118 | 1 |
|  | **Nem vs SO4** | 1 | 0.387 | 1.01 | 0.091 | 0.427 | 1 | 1 | 0.312 | 1.03 | 0.093 | 0.421 | 1 |
|  | **Nem vs RGM** | 1 | 0.455 | 1.32 | 0.117 | 0.187 | 1 | 1 | 0.216 | 0.85 | 0.079 | 0.623 | 1 |
|  | **Nem vs 41B** | 1 | 0.657 | 1.90 | 0.160 | 0.014 | 0.21 | 1 | 0.569 | 1.59 | 0.137 | 0.067 | 1 |
|  | **Nem vs 3309C** | 1 | 0.563 | 1.94 | 0.163 | 0.026 | 0.39 | 1 | 0.264 | 0.97 | 0.089 | 0.442 | 1 |
|  | **SO4 vs RGM** | 1 | 0.490 | 1.31 | 0.116 | 0.151 | 1 | 1 | 0.279 | 1.01 | 0.092 | 0.488 | 1 |
|  | **SO4 vs 41B** | 1 | 0.574 | 1.53 | 0.132 | 0.061 | 0.915 | 1 | 0.322 | 0.84 | 0.078 | 0.594 | 1 |
|  | **SO4 vs 3309C** | 1 | 0.431 | 1.34 | 0.119 | 0.15 | 1 | 1 | 0.452 | 1.53 | 0.132 | 0.163 | 1 |
|  | **RGM vs 41B** | 1 | 0.325 | 0.97 | 0.089 | 0.52 | 1 | 1 | 0.563 | 1.69 | 0.144 | 0.048 | 0.72 |
|  | **RGM vs 3309C** | 1 | 0.708 | 2.54 | 0.202 | 0.004 | 0.06 | 1 | 0.548 | 2.22 | 0.182 | 0.037 | 0.555 |
|  | **41B vs 3309C** | 1 | 0.938 | 3.34 | 0.250 | 0.003 | **0.045** | 1 | 0.665 | 1.89 | 0.159 | 0.032 | 0.48 |
| **Scion** | **CS vs PN** | 1 | 0.452 | 1.22 | 0.108 | 0.215 | 1 | nd | | | | | |
|  | **CS vs UB** | 1 | 0.466 | 1.33 | 0.117 | 0.185 | 1 | nd | | | | | |
|  | **CS vs Syr** | 1 | 0.782 | 2.79 | 0.218 | 0.013 | 0.13 | nd | | | | | |
|  | **CS vs Gre** | 1 | 0.315 | 0.90 | 0.082 | 0.554 | 1 | nd | | | | | |
|  | **PN vs UB** | 1 | 0.688 | 1.77 | 0.150 | 0.011 | 0.11 | nd | | | | | |
|  | **PN vs Syr** | 1 | 0.866 | 2.72 | 0.213 | 0.014 | 0.14 | nd | | | | | |
|  | **PN vs Gre** | 1 | 0.473 | 1.21 | 0.108 | 0.21 | 1 | nd | | | | | |
|  | **UB vs Syr** | 1 | 0.930 | 3.13 | 0.238 | 0.009 | 0.09 | nd | | | | | |
|  | **UB vs Gre** | 1 | 0.482 | 1.31 | 0.116 | 0.163 | 1 | nd | | | | | |
|  | **Syr vs Gre** | 1 | 0.455 | 1.52 | 0.132 | 0.109 | 1 | nd | | | | | |

**Table S8** Comparison of the abundances of the bacterial metabolic pathways predicted with PICRUST2 between the rhizosphere and the root endosphere.

Asterisks represent significant differences between the root system compartments using Student or Wilcoxon test: *P<0.05, **P<0.01, ***P<0.001, n=60.

| **Metabolic pathways** | **Rhizosphere** | | **Root endosphere** | | ***p*-value** | |
| --- | --- | --- | --- | --- | --- | --- |
|  | **mean** | **sd** | **mean** | **sd** |  |  |
| **Amino acid metabolism** | 132939 | 2008 | 131251 | 2278 | **3.72e-05** | ******* |
| **Biosynthesis of other secondary metabolites** | 26355 | 1888 | 23129 | 1858 | **5.923e-16** | ******* |
| **Carbohydrate metabolism** | 135468 | 2329 | 130675 | 1864 | **2.438e-16** | ******* |
| **Energy metabolism** | 50142 | 910 | 45456 | 1894 | **< 2.2e-16** | ******* |
| **Glycan biosynthesis and metabolism** | 24839 | 1135 | 22280 | 2688 | **7.392e-08** | ******* |
| **Lipid metabolism** | 57421 | 1571 | 65740 | 4302 | **< 2.2e-16** | ******* |
| **Membrane transport** | 17916 | 1438 | 15387 | 1517 | **8.05e-16** | ******* |
| **Metabolism of cofactors and vitamins** | 110981 | 2650 | 101894 | 5369 | **< 2.2e-16** | ******* |
| **Metabolism of other amino acids** | 49166 | 1735 | 49682 | 2586 | **0.004209** | ****** |
| **Metabolism of terpenoids and polyketides** | 92957 | 3598 | 91588 | 4873 | **0.01689** | ***** |
| **Nucleotide metabolism** | 13917 | 371 | 12766 | 548 | **< 2.2e-16** | ******* |
| **Signal transduction** | 4844 | 586 | 3898 | 313 | **6.131e-16** | ******* |
| **Signaling molecules and interaction** | 7 | 6 | 3 | 8 | **5.289e-07** | ******* |
| **Xenobiotics biodegradation and metabolism** | 83738 | 7064 | 124334 | 11289 | **< 2.2e-16** | ******* |

**Table S9** Comparison of the abundances of the bacterial metabolic pathways predicted with PICRUST2 between rootstock or scion genotypes in the rhizosphere or the root endosphere.

Significances were assessed through a Two-way ANOVA. Asterisks represent significant effects *P<0.05, **P<0.01, ***P<0.001

|  |  | **Influence of the rootstock** | | | | | | **Influence of the scion** | | | | | |
| --- | --- | --- | --- | --- | --- | --- | --- | --- | --- | --- | --- | --- | --- |
|  | | **Rhizosphere** | | | **Root endosphere** | | | **Rhizosphere** | | | **Root endosphere** | | |
|  |  | ***p*-value** | | **PVE** | ***p*-value** | | **PVE** | ***p*-value** | | **PVE** | ***p*-value** | | **PVE** |
| **Amino acid metabolism** | **Genotype (G)** | **8.00e-10** | ******* | **80%** | **0.002** | ****** | **46%** | 0.088 | ns | / | 0.917 | ns | / |
|  | **Block (B)** | 0.101 | ns | / | 0.493 | ns | / | 0.891 | ns | / | 0.247 | ns | / |
|  | **G x B** | 0.194 | ns | / | 0.144 | ns | / | 0.103 | ns | / | **0.029** | ***** | **38%** |
| **Biosynthesis of other secondary metabolites** | **G** | **4.40e-04** | ******* | **49%** | 0.996 | ns | / | 0.278 | ns | / | 0.337 | ns | / |
|  | **B** | 0.073 | ns | / | 0.597 | ns | / | 0.198 | ns | / | 0.051 | ns | / |
|  | **G x B** | 0.114 | ns | / | 0.971 | ns | / | 0.928 | ns | / | 0.477 | ns | / |
| **Carbohydrate metabolism** | **G** | **3.89e-04** | ******* | **51%** | 0.396 | ns | / | **0.037** | ***** | **33%** | 0.115 | ns | / |
|  | **B** | 0.230 | ns | / | 0.414 | ns | / | 0.719 | ns | / | 0.111 | ns | / |
|  | **G x B** | 0.170 | ns | / | 0.284 | ns | / | **0.048** | ***** | **24%** | 0.440 | ns | / |
| **Energy metabolism** | **G** | **0.016** | ***** | **18%** | 0.528 | ns | / | **0.039** | ***** | **28%** | 0.267 | ns | / |
|  | **B** | **1.64e-07** | ******* | **51%** | 0.811 | ns | / | **0.023** | ***** | **14%** | 0.269 | ns | / |
|  | **G x B** | **0.018** | ***** | **13%** | 0.520 | ns | / | **0.004** | ****** | **30%** | 0.889 | ns | / |
| **Glycan biosynthesis and metabolism** | **G** | **1.33e-05** | ******* | **58%** | 0.380 | ns | / | 0.075 | ns | / | 0.949 | ns | ns |
|  | **B** | **0.016** | ***** | **8%** | 0.243 | ns | / | 0.367 | ns | / | **0.014** | ***** | **23%** |
|  | **G x B** | 0.360 | ns | / | 0.630 | ns | / | 0.109 | ns | / | **0.006** | ****** | **39%** |
| **Lipid metabolism** | **G** | **4.81e-06** | ******* | **64%** | **0.013** | ***** | **38%** | **0.008** | ****** | **42%** | 0.805 | ns | / |
|  | **B** | 0.15 | ns | / | 0.670 | ns | / | 0.467 | ns | / | 0.253 | ns | / |
|  | **G x B** | 0.340 | ns | / | 0.709 | ns | / | 0.074 | ns | / | **0.006** | ****** | **45%** |
| **Membrane transport** | **G** | **2.47e-04** | ******* | **51%** | **0.002** | ****** | **46%** | **0.026** | ***** | **36%** | 0.530 | ns | / |
|  | **B** | 0.075 | ns | / | 0.721 | ns | / | 0.625 | ns | / | 0.778 | ns | / |
|  | **G x B** | 0.306 | ns | / | 0.381 | ns | / | 0.119 | ns | / | **0.047** | ***** | **34%** |
| **Metabolism of cofactors and vitamins** | **G** | **0.016** | ***** | **31%** | 0.870 | ns | / | 0.119 | ns | / | 0.690 | ns | / |
|  | **B** | **0.005** | ****** | **17%** | 0.743 | ns | / | 0.077 | ns | / | 0.075 | ns | / |
|  | **G x B** | 0.230 | ns | / | 0.198 | ns | / | **0.013** | ***** | **31%** | **0.008** | ****** | **40%** |
| **Metabolism of other amino acids** | **G** | **5.52e-04** | ******* | **47%** | 0.498 | ns | / | 0.056 | ns | / | 0.112 | ns | / |
|  | **B** | **0.019** | ***** | **9%** | 0.335 | ns | / | 0.465 | ns | / | 0.332 | ns | / |
|  | **G x B** | 0.123 | ns | / | 0.668 | ns | / | 0.151 | ns | / | **0.013** | ***** | **33%** |
| **Metabolism of terpenoids and polyketides** | **G** | **3.28e-04** | ******* | **42%** | 0.089 | ns | / | 0.137 | ns | / | 0.690 | ns | / |
|  | **B** | **3.40e-04** | ******* | **21%** | 0.089 | ns | / | **0.0220** | ***** | **16%** | **0.021** | ***** | **19%** |
|  | **G x B** | 0.051 | ns | / | 0.267 | ns | / | 0.421 | ns | / | **0.007** | ****** | **37%** |
| **Nucleotide metabolism** | **G** | **0.002** | ****** | **39%** | 0.966 | ns | / | 0.069 | ns | / | 0.842 | ns | / |
|  | **B** | **0.003** | ****** | **16%** | 0.461 | ns | / | 0.407 | ns | / | 0.095 | ns | / |
|  | **G x B** | 0.460 | ns | / | 0.400 | ns | / | 0.061 | ns | / | **0.019** | ***** | **38%** |
| **Signal transduction** | **G** | **1.50e-04** | ******* | **53%** | **3.67e-05** | ******* | **60%** | **0.030** | ***** | **35%** | 0.379 | ns | / |
|  | **B** | 0.080 | ns | / | 0.846 | ns | / | 0.680 | ns | / | 0.164 | ns | / |
|  | **G x B** | 0.222 | ns | / | 0.257 | ns | / | 0.119 | ns | / | 0.698 | ns | / |
| **Signaling molecules and interaction** | **G** | **0.002** | ****** | **42%** | 0.681 | ns | / | 0.078 | ns | / | 0.574 | ns | / |
|  | **B** | **0.023** | ***** | **10%** | 0.217 | ns | / | 0.837 | ns | / | 0.553 | ns | / |
|  | **G x B** | 0.062 | ns | / | 0.454 | ns | / | **0.002** | ****** | **40%** | 0.451 | ns | / |
| **Xenobiotics biodegradation and metabolism** | **G** | **0.013** | ***** | **25%** | 0.484 | ns | / | **0.020** | ***** | **33%** | 0.167 | ns | / |
|  | **B** | **4.94e-05** | ******* | **33%** | 0.764 | ns | / | **0.028** | ***** | **12%** | 0.147 | ns | / |
|  | **G x B** | 0.296 | ns | / | 0.562 | ns | / | 0.050 | ns | / | 0.104 | ns | / |
